# Genomic Insights Into the Evolutionary History of *Berardius* Beaked Whales: Speciation Driven by Resource Specialization, Gigantism and Thermal Barriers?

**DOI:** 10.1111/mec.70426

**Published:** 2026-06-17

**Authors:** Morgan L. McCarthy, Michael V. Westbury, Mark S. Springer, John Gatesy, Morten Tange Olsen, Phillip A. Morin

**Affiliations:** ^1^ Globe Institute University of Copenhagen Copenhagen K Denmark; ^2^ Department of Population Analysis and Monitoring Swedish Museum of Natural History Stockholm Sweden; ^3^ Department of Health Technology, Section for Bioinformatics Technical University of Denmark Kongens Lyngby Denmark; ^4^ Department of Evolution, Ecology, and Organismal Biology University of California, Riverside Riverside California USA; ^5^ Division of Vertebrate Zoology American Museum of Natural History New York New York USA; ^6^ Southwest Fisheries Science Center, National Marine Fisheries Service, NOAA La Jolla California USA

**Keywords:** demographic history, introgression, marine diversity, phylogenomics, speciation, Ziphiidae

## Abstract

Understanding the diversity of our oceans is fundamental now more than ever as climate change and human activities put increasing pressure on marine species and ecosystems. Beaked whales (family Ziphiidae) are among the most poorly understood marine mammals, in part due to their affinity to offshore underwater canyons and prolonged diving ability. The ziphiid genus *Berardius* is comprised of three extant species; however, a lack of genomic data results in uncertainties of their evolutionary origin, phylogenetic relationships and the past environmental drivers of speciation and demographic fluctuations. Here, we analyse whole nuclear and mitochondrial genomes to better understand the evolutionary and demographic history of *Berardius* spp. Genome‐wide comparisons support an initial divergence of *B. minimus* from the *B. bairdii* – *B. arnuxii* common ancestor ~4 million years ago (mya), and a divergence between *B. bairdii* and *B. arnuxii* ~1.5 mya. Despite these deep divergence times, gene flow was detected between species pairs for at least 75% of their post‐divergence branch lengths. Strong levels of introgression (~8%) were detected between the non‐sister but geographically overlapping *B. bairdii* and *B. minimus* species, despite their significant differences in body size. Our results show that the *Berardius* speciation process goes beyond simple bifurcating divergences. We hypothesize that resource specialization and an increased body size in the *B. bairdii* – *B. arnuxii* ancestor may have been an incipient driver of speciation and further that climate cycles during the late Pleistocene, coupled with ranging movements to the tropics, could have permitted repeated opportunities for gene flow between species.

## 1 Introduction

The terrestrial environment is characterized by spatial variation in climate, topography and habitat distribution which all contribute to the formation of new species (Costello and Chaudhary 2017). In contrast, in the ocean, there are few physical barriers to dispersal, yet new species continue to originate, and many marine species occur in discrete subspecies, populations and ecotypes. This phenomenon is known as the marine‐speciation paradox (Bierne et al. 2003) and has drawn considerable academic interest (Faria et al. 2021; Wang et al. 2021; Westbury et al. 2023); all seeking to inform the question of how speciation occurs in the absence of obvious barriers to dispersal.

Beaked whales (Ziphiidae) inhabit the ocean's pelagic zone, with almost no geographic barriers to dispersal, and yet, they are the second most speciose family of the order Cetacea, numbering (currently) 24 species across six genera. Their preference for deep‐sea squid and fish restricts them to habitats between the continental slope and abyssal plain, with a strong preference for submarine canyons (Waring et al. 2001; Ferguson et al. 2005). The amount of time beaked whales spend below the surface combined with their presumed naturally low abundance makes gathering biological data difficult and time consuming. One long‐term study reported 3.5 beaked whale sightings for every 100 h of search effort (Baird 2019) and some species of beaked whales are known only from a handful of unconfirmed sightings or beach‐cast specimens (Carwardine 2019). Thus, most beaked whale species have poorly described biology, distribution and demographic history. The International Union for Conservation of Nature (IUCN) Red List reports 8 of 24 assessed species of beaked whales as data deficient and 21 lack any indication of population trend (IUCNredlist.org, consulted 6 May 2026). In light of the many difficulties associated with studying beaked whales, researchers have turned to molecular methods. The application of genetic methods has been fundamental in resolving taxonomy, describing new species and inferring the evolutionary and demographic history of beaked whales (Dalebout et al. 2002, 2004, 2008, 2014; Feyrer et al. 2019; Yamada et al. 2019), but genome‐scale studies of species in this clade are just beginning to emerge (Carroll et al. 2021; Westbury et al. 2021; de Greef et al. 2022; Onoufriou et al. 2022).

**FIGURE 1:**
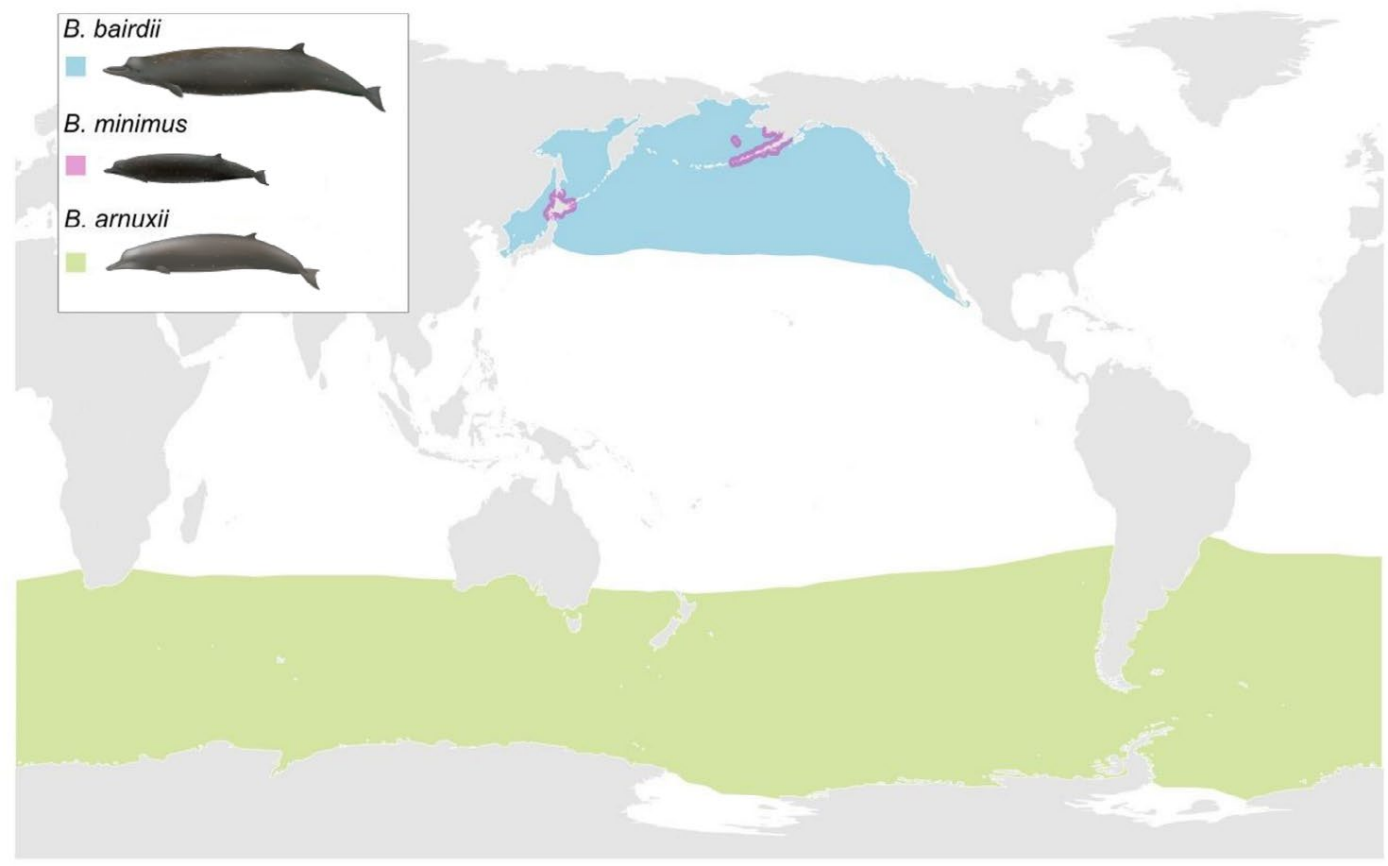
Global map illustrating the assumed range of each of the three *Berardius* species shown in the key. The ranges of *B. bairdii* and *B. arnuxii* were based on IUCN assessments (IUCN 2020a, 2020b) and from Yamada et al. (2019) for *B. minimus* . Species illustrations were reprinted from Thewissen (2018). Illustrations are scaled by maximum recorded body size as reported in Carwardine (2019).

The genus *Berardius* is sister to all other ziphiids (McGowen et al. 2020) and consists of three species (Figure 1). These differ in their distribution, with Baird's beaked whale ( *B. bairdii* ) occurring across the North Pacific, Sato's beaked whale ( *B. minimus* ) so far only reported in a small range of the North Pacific, and Arnoux's beaked whale ( *B. arnuxii* ) with a wider distribution across the southern hemisphere (Omura et al. 1955; Kitamura et al. 2013; Morin et al. 2017; Yamada et al. 2019). Although the two North Pacific species ( *B. minimus* and *B. bairdii* ) were already suggested to be separate forms in the 1950s (Omura et al. 1955), and subsequent analyses of mitochondrial DNA data indicated the presence of cryptic diversity within *Berardius* (Kitamura et al. 2013; Morin et al. 2017), the existence of *B. minimus* was not formally recognized until recently in a combined analysis of genetic and morphological data (Yamada et al. 2019). *B. minimus* differs from *B. bairdii* and *B. arnuxii* by having a much smaller mature body size, a proportionately shorter beak, and a darker blackish body colour. Notably, *B. bairdii* and *B. arnuxii* are giants among the beaked whale clade, while *B. minimus* has a more typical body size for a beaked whale.

Yet, while recognized as three separate species, their evolutionary history remains unresolved, and little is known with regard to the timing and drivers of speciation. Furthermore, the large differences in distribution and body size of the three species make them an interesting case study to explore the marine speciation paradox in a beaked whale context. Before the formal description of *B. minimus* , it was suggested that the North Pacific *B. bairdii* and southern hemisphere *B. arnuxii* arose allopatrically, separated by warm tropical waters (Steeman et al. 2009). More recently, it has been suggested that an initial species divergence resulted in *B. minimus* in the North Pacific and the ancestor of *B. bairdii* and *B. arnuxii* in the southern hemisphere, with a subsequent dispersal of *B. bairdii* from the southern hemisphere to the North Pacific (Morin et al. 2017). While this hypothesis is consistent with mitogenome data (Kitamura et al. 2013; Morin et al. 2017; Yamada et al. 2019), a nuclear DNA phylogeny based on the first intron of nuclear α‐2‐actin gene ACTA2I gene indicated that *B. arnuxii* is sister to the other two extant *Berardius* species, but support for this relationship was weak (Kitamura et al. 2013).

Here, we present analyses of complete nuclear and mitochondrial genomes to shed light on the evolutionary history of *Berardius* beaked whales. Specifically, we reconstruct the phylogenetic relationships and divergence times among the three *Berardius* species and related beaked and toothed whales. We also estimate levels of genetic diversity and differentiation and infer introgression (gene flow) patterns. Finally, we provide insights into the demographic histories of all three species and present hypotheses on the environmental factors that may have shaped their evolutionary history. Our data and findings provide an essential resource for understanding marine speciation processes, adaptations to deep‐diving, and delineating beaked whale species, populations and management units.

## 2 Methods

### 2.1 Samples, DNA Extraction and Sequencing

Tissue samples for *B. minimus* (*n* = 2), *B. arnuxii* (*n* = 1) and *B. bairdii* (*n* = 2) were collected from beach‐cast or floating carcasses and stored in salt‐saturated 20% DMSO at −20°C at the U.S. National Marine Fisheries Service (NMFS) Marine Mammal and Sea Turtle Research (MMASTR) Collection at the Southwest Fisheries Science Center (SWFSC; Table S1). DNA was extracted at the SWFSC using a sodium chloride precipitation protocol (Miller et al. 1988) and sent to the Genomics Core facility at the University of California, Riverside for library preparation on an Illumina Neo‐Prep machine and sequenced using Illumina HiSeq 2500 (150 bp paired‐end reads) at the New York Genome Center (NYGC). The initial extract for *B. bairdii* (z0017152) yielded a low depth of coverage; thus, a second extract from a new sample (z0076728) was sequenced in the same manner. To further increase the depth of coverage of the second sample, DNA extract was also sent to GENEWIZ (South Plainfield, NJ, USA) for library preparation and sequencing with Illumina HiSeq 2000 (150 bp PE). The sequencing data for the second *B. bairdii* sample from NYGC and GENEWIZ were combined following quality control and adapter trimming. The small number of genomes available per species represents a limitation of this study and reflects the difficulty of obtaining high‐quality samples from rare and cryptic taxa. Although whole‐genome approaches can recover informative signals of historical demography and divergence from few individuals, particularly at deeper timescales, such analyses cannot account for unknown intraspecies population structure. As a result, our analyses are restricted to broad‐scale demographic patterns and relative divergence timing. Increasing sample sizes across the respective ranges for all three *Berardius* species should be a future research priority to further inform the results presented in this study.

### 2.2 Data Processing and Mapping

Raw data processing and mapping of the mitochondrial genome (mitogenome) and whole nuclear genome followed protocols adapted from Morin et al. (2018). Adapters were first trimmed from reads using BBDuk (https://sourceforge.net/projects/bbmap/) with the parameters ktrim = *r*, k = 23, mink = 8, hdist = 1, tbo, qtrim = rl, trimq = 15, maq = 20, minlen = 40. The linearized *B. bairdii* reference mitogenome (NCBI Accession ID: AJ554057) was prepared for mapping by ‘padding’ ends with 40 base pairs (bp) of the opposite end. This step was taken to increase coverage across the artificial break of the circular mitogenome. Reads from paired‐end libraries were then mapped to the *B. bairdii* mitogenome using Burrows‐Wheeler Aligner Maximal Exact Matches (BWA‐MEM; Li and Durbin 2009) and default parameters. PCR duplicates were removed with SAMtools v.1.9 (Li et al. 2009). Consensus sequences were called from mapped bam files in ANGSD (Korneliussen et al. 2014) with the highest effective depth option (‐dofasta 3) and filters for minimum mapping quality of 25, minimum base quality of 25, reads that mapped only to one unique location on the reference genome (‐uniqueonly) and for sites with a minimum read depth of three (‐minindepth 3). The mapping process was repeated using the new reference mitogenome for each species to minimize chances of sequence assembly bias (Prasad et al. 2022). An additional *B. arnuxii* mitogenome was assembled from raw reads (ENA accession ID: SRR10251437) generated in McGowen et al. (2020). The raw reads were trimmed and mapped as previously described for the *B. arnuxii* mitogenome generated in this study. The reads representing the nuclear genome data were mapped with BWA‐MEM to a platinum‐standard reference‐quality (Morin et al. 2025) Blainville's beaked whale ( *Mesoplodon densirostris* : GCA_025265405.1) genome. For a subset of downstream analyses, repetitive regions assessed with RepeatMasker v.4.1.5 (Smit et al. 2013) were masked from mapped BAM files with BEDtools v.2.29 (Quinlan and Hall 2010).

### 2.3 Fossil and Molecular Calibrated Nuclear Genome Phylogeny

We constructed a nuclear genome phylogeny using our novel *Berardius* genome data, as well as four additional odontocete genome assemblies including the killer whale ( *Orcinus orca* ; Delphinidae), vaquita ( *Phocoena sinus* ; Phocoenidae), beluga whale ( *Delphinapterus leucas* ; Mondodontidae) and Gervais's beaked whale ( *Mesoplodon europaeus* ; Ziphiidae), which were downloaded from GenBank and DNAzoo (https://www.dnazoo.org/) (Table S2). These outgroup genomes were selected for their high BUSCO gene completeness scores and their representation of several families across Odontoceti. Consensus sequences for the higher depth of coverage *B. minimus* sample (z0041749), *B. bairdii* (z0076728) and *B. arnuxii* were called from the mapped bam files using the ANGSD v.0.931 ‐doFasta 2 command and included the filters: minimum mapping score of 25, minimum base quality score of 30. The presence of 12,536 single copy candidate orthologs contained in the Cetartiodactyla Orthodb database were assessed for the seven genomes through the BUSCO v.4. pipeline (Simão et al. 2015) using gVolante2 (Nishimura et al. 2017, 2019). The 8639 orthologs that were evaluated as complete across all seven genomes were retained for further downstream analyses and were separately aligned with the codon aware aligner PRANK (Löytynoja 2013). Gene alignments (*n* = 953) flagged by PRANK as having codon sequence lengths not divisible by three were omitted as poorly aligned genes. Gaps with missing data across the remaining 7686 gene alignments were pruned with GBLOCKS including the options ‐t = c ‐b5 = h ‐d = y ‐*p* = y (https://www.biologiaevolutiva.org/jcastresana/Gblocks.html). To further screen gene alignments for excessive mismatches, likely the result of poor mapping or low coverage regions, a custom R script (available on GitHub) was used to screen average pairwise distances between a sequence and all others in an alignment, and those sequences with an average pairwise distance > 10% were discarded from the alignment. We omitted gene alignments where any sequence was discarded for excessive mismatches, resulting in 7534 remaining gene alignments. Finally, to account for the impact incomplete lineage sorting or gene flow between lineages could have on estimating divergence times, individual gene alignments and sites within those genes were screened to determine if they were concordant with the species tree. To do this we first computed individual gene trees for each locus using IQ‐tree v.1.6.12 (Nguyen et al. 2015) and inferred the overall species tree under a multi‐species coalescence model, using ASTRAL v.5.7.8 (Zhang et al. 2018) with the gene trees as input. Gene trees that were topologically discordant to the species tree or had mean bootstrap values < 90 were removed. The remaining gene alignments were subsequently concatenated with FASconCAT‐G ‐a ‐p ‐p ‐s ‐l (https://github.com/PatrickKueck/FASconCAT‐G). This resulted in a final alignment of 695,592 bp for the dated phylogeny. A maximum likelihood phylogeny was created from the alignment using IQ‐tree with the GTR substitution model and 10,000 ultrafast bootstrap (Minh et al. 2013) replicates. Three node calibrations based on fossil dates for a crown odontocete, crown ziphiid and the split between Phocoenidae (porpoises) and Monodontidae (narwhal, beluga) were included (Table S2). Following Dos Reis and Yang (2019), MCMCtree was used to estimate species divergence times with three independent runs, an 80,000 chain burnin and 40 M iterations. The three independent runs were checked for convergence and output files were visualized in Tracer (http://tree.bio.ed.ac.uk/software/tracer/) and in R v.3.6.3 (R Core Team 2019) with ggtree v.2.0.4 (Yu et al. 2017).

### 2.4 Tree Topology Sliding Window Discordance

We generated consensus autosomal pseudo‐haploid base calls (‐dohaplocall 2) for single individual representatives from the *Berardius* spp. and the outgroup *M. europaeus* , using the higher coverage representative of *B. minimus* (z0041749) and of *B. bairdii* (z0076728), using ANGSD v.0.935 and the following parameters: considering only reads with a minimum mapping score of 30, minimum base quality score of 30, a minimum site depth of coverage per genome of 10, the GATK genotype likelihood model, calculated the major and minor allele using the genotype likelihoods, considered only the autosomes, removed reads that mapped to multiple places on the reference genome, only considered sites for which genotype likelihoods could be called for a minimum of four samples, and sites with more than 1 minor sampled allele across samples. From the pseudo‐haploid file, we computed sliding window phylogenies in PhyML v.3.3.20211231 (Guindon et al. 2010) using an available python script at https://github.com/simonhmartin/genomics_general (phyml_sliding_windows.py). We specified a window size of 20 kb, a slide of 1 Mb to account for linkage disequilibrium, a window must contain at least 20 variable sites, and PhyML to optimize the following parameters: tree topology (t), branch length (l) and substitution rate parameters (r). We counted the number of window trees for each of the potential quartets using ASTRAL and the parameter ‐t 32.

### 2.5 Genome‐Wide and Sliding Window Genetic Distances

Genome‐wide and sliding window genetic distances were calculated for *Berardius* and outgroup beaked whales to inform and contextualize the evolutionary distances within *Berardius* beaked whales. Raw reads (Table S2) for Sowerby's beaked whale ( *Mesoplodon bidens* ), Blainville's beaked whale, Gervais' beaked whale, Stejneger's beaked whale ( *Mesoplodon stejnegeri* ) and the goose‐beaked whale ( *Ziphius cavirostris* ) were downloaded from ENA and mapped as described above to the *Mesoplodon densirostris* reference genome. Following the removal of reads mapped to repetitive regions in mapped BAM files, genome‐wide pairwise distances for all species pairs were calculated using a consensus base call in ANGSD (‐doIBS 2), considering only sites with a minimum depth of 10 and the filters: minimum mapping quality (30), minimum base quality score (30) and reads that uniquely mapped to one region of the reference genome. This analysis was limited to the 20 autosomal chromosomes. Another calculation of sliding window distances was conducted using just the *Berardius* species and repeated separately for the autosomes and allosomes (sex chromosomes). Non‐overlapping windows of 1 Mb and 100 kb were prepared from the *M. densirostris* reference genome using BEDtools v.2.29. The significance of average distances between species pairs was assessed using a two‐sample Kolmogorov–Smirnov test implemented in base R. This was done separately for autosomes and allosomes.

### 2.6 Determining Gene Flow Intervals: Hybrid PSMC

To infer end of gene flow between the three *Berardius* species, consensus sequences of the 20 autosomes were called (‐dofasta 3) from mapped bam files in ANGSD with the highest effective depth option and filters for minimum mapping quality of 25, minimum base quality of 25, reads that mapped only to one unique location on the reference genome and for sites with a minimum read depth of three. Hybrid PSMC input files were generated from the consensus sequences with the default settings of the pscmfa_from_2_fastas.py script provided by Cahill et al. (2016). PSMC was implemented with the settings ‐N25 ‐t15 ‐r5 ‐p “4 + 25*2 + 4 + 6” and visualized with the plot_psmc.pl script from (Li and Durbin 2011) using a generalized *Berardius* generation time of 25.9 years (Taylor et al. 2007) and a mutation rate of 1.15e‐8 substitutions/site/generation averaged from killer whale and bottlenose dolphin ( *Tursiops truncatus* ) germline mutation rate estimates (Bergeron et al. 2023). A pre‐divergence effective population size (*N*_e_) for further simulations was inferred by visually inspecting the text file output and choosing the value just before *N*_e_ steeply increased and later updated to ensure simulation and empirical *N*_e_ converged. Upper and lower range estimates for end of gene flow were simulated with ms (Hudson 2002) as in Cahill et al. (2016), using 50,000‐year intervals and the inferred pre‐divergence *N*_e_ as input. The output was visualized in R and the simulations surrounding but not overlapping the empirical iteration within the range of 1.5 × *N*_e_ and 10 × *N*_e_ were selected as the minimum and maximum end of gene flow bounds.

### 2.7 Admixture Inference With *D*‐Statistics: ABBA‐BABA

To further assess evidence of admixture after the initial divergence of the three *Berardius* species, we used the ABBA‐BABA test (also referred to as the *D*‐statistic) (Durand et al. 2011; Green et al. 2010; Hibbins and Hahn 2021), followed by the f4 admixture ratio and the f‐branch statistic (Malinsky et al. 2018), implemented in Dsuite v.0.5.r53 (Malinsky et al. 2021). The ABBA‐BABA test counts the genome‐wide occurrence of derived alleles across groups of three species (trios) and an outgroup for inference of alternative species trees that indicate genetic introgression. The f4‐ratio is expected to be linear in relation to the proportion of introgression. Because trios are not independent, the f‐branch statistic was used to infer the position of gene flow on the tree by identifying excess sharing of derived alleles between branches and species on a tree (Malinsky et al. 2018).

We implemented the ABBA‐BABA test using repeat masked mapped bam files and *Mesoplodon densirostris* as an outgroup, with the ‐doAbbababa 2 option in ANGSD and filters: minimum read depth per site (10), minimum quality score (30), minimum mapping score (30), removing reads flagged by angsd (‐remove bads), including reads that mapped uniquely to one spot on the reference genome and using block sizes of 1 Mb (‐blocksize 1,000,000), and limited the analysis to just the autosomes. The output was analysed with the Rscript jackKnife.R (https://github.com/ANGSD/angsd/blob/master/R/jackKnife.R) to perform jackknife bootstraps and calculate the *D*‐statistic and |*Z*‐score|. A *Z*‐score > |3| was considered significant.

Dsuite uses a VCF file as input, so we converted the previously generated repeat‐masked *Berardius* species, *Mesoplodon europaeus* and *Phocoena sinus* bam files to VCF files using BCFtools v.1.11 (Danecek et al. 2021) with minimum base and map quality of 30. The individual VCF files of autosome and X chromosome scaffolds were merged and further filtered for sites with quality > 30 and depth > 10. Using the tree established in our phylogenomic analysis, we calculated *D*‐statistics, f4‐ratio and f‐branch statistics for the topology ((( *Berardius bairdii* , *Berardius arnuxii* ) *Berardius minimus* )Outgroup), with *M. europaeus* as the outgroup.

### 2.8 Historical Demography (PSMC)

Diploid consensus sequences from the *Berardius* species, excluding the lower coverage *B. minimus* (z0007969) and *B. bairdii* (z0017152) genomes were generated for PSMC v.0.6.5 (Li and Durbin 2011) using the bcftools v.1.9 (Danecek et al. 2021) commands bcftools mpileup to create a vcf, calling genotypes with bcftools call, considering minimum and maximum depth cut‐offs of one‐third and twice the average depth of coverage for each genome respectively, and converting the output to a fastq file with the vcfutils.pl script. The fastq files were converted to a psmcfa file with the fq2psmcfa script provided in the psmc/utils folder. PSMC was implemented with the settings ‐N25 ‐t15 ‐r5 ‐p “2 + 2 + 25*2 + 4 + 6”. The psmc files were processed with the plot_psmc.pl script and scaled with a generalized *Berardius* species generation time of 25.9 years and mutation rate of 1.15e‐8 substitutions/site/generation.

### 2.9 Genome‐Wide Heterozygosity Estimates

Genome‐wide heterozygosity estimates were calculated for autosomal chromosomes using ANGSD for *M. bidens* , *M. densirostris* , *M. europaeus* , *M. stejnegeri* and *Z. cavirostris* . Sample allele frequencies for the autosomes were generated with the ANGSD ‐doSaf 1 command and the filters ‐setMinDepthInd 10, ‐minmapq 30, ‐minq 30, ‐uniqueonly 1, ‐only_proper_pairs 1. Heterozygous sites were quantified using realSFS implemented in ANGSD and visualized in R. The impact of repetitive regions on the heterozygosity estimates was explored by rerunning the analysis with repeat‐masked mapped bam files. To verify heterozygosity estimates using the above method, we used a modified version of a previously published pipeline that utilizes allele counts, which is less prone to the effects of DNA damage and sequencing errors (Hempel et al. 2024). In brief, we used ANGSD to directly perform allele counts on the autosomes of the mapped non‐repeat masked bam files using the following command and filters: ‐minQ 20 ‐minMapQ 20 ‐uniqueOnly 1 ‐remove_bads 1 ‐doCounts 1 ‐dumpCounts 4. We set the minimum depth as half the mean coverage of the given individuals. We only called heterozygous sites if the minor allele was > 5%. We visualized all resultant minor allele frequencies for heterozygous base calls and chose a 25% minor allele frequency threshold to call a site as heterozygous. We reran the analysis with a 25% threshold to call a site as heterozygous. That is, a site with an allele found at a frequency of > 75% was called homozygous, and any site that had two alleles with a frequency of > 25% each was called heterozygous. Sites not meeting these criteria were counted but not given a base call. We divided the total number of heterozygous bases by the total number of sites that passed the initial ANGSD filtering.

### 2.10 Mitogenome Phylogenetics and Haplotype Network

To supplement the nuclear genome analyses, we inferred the mitogenome phylogenetic relationships among *Berardius* and related toothed whales, including representative mitogenomes from Ziphiidae, Kogiidae, Delphinidae, Monodontidae and Phocoenidae (Table S3). Mitogenomes were aligned with MAFFT v. 7.388 (Katoh et al. 2002; Katoh and Standley 2013) and the 13 protein‐coding genes and 2 rRNA genes manually extracted from the alignment with Geneious Prime 2020.1.2 (https://www.geneious.com/). Mitogenome regions were concatenated into a single sequence after removing stop codons from protein‐coding genes and indels from rRNA segments. The overlapping region for ATP6 and ATP8 was removed due to inconsistencies in the number of overlapping nucleotides between species. Substitution models for two RNA and 13 coding regions, including 1st, 2nd and 3rd codon positions for the coding regions, were established with PartitionFinder v.2.1.1 (Lanfear et al. 2017) and resulted in a partitioning scheme with eight subsets, each with an independent substitution model (Table S4). A maximum likelihood tree was inferred using IQ‐tree and support determined using 10,000 ultrafast bootstraps. The consensus tree was rooted to the outgroup *Kogia* spp. and visualized with R and the ggtree package.

Species divergence times were estimated using a Bayesian phylogenetic approach implemented in BEAST v.2.6.3 (Bouckaert et al. 2019). A relaxed log normal clock was applied and the Yule tree model was calibrated with the age of *Ferecetotherium kelloggi* (Mchedlidze 1970; log normal distribution—offset: 23 Mya, M: 1.0, S: 1.0) as the calibration date for the odontocete crown clade and *Archaeoziphius microglenoideus* (log normal distribution—offset: 13.2 Mya, M: 1.0, S: 1.0) for the crown Ziphiidae lineage (Lambert and Louwye 2006). The model was run three independent times with 50,000,000 generations. The log files were combined with LogCombiner v.2.6.6 (distributed with BEAST) and inspected in Tracer after a 20% burnin for each run. All parameters had ESS values > 200. The tree files were similarly combined with LogCombiner after a 20% burnin of each tree file and a Maximum Clade Credibility (MCC) tree was prepared in TreeAnnotator v.2.6.3 (Bouckaert et al. 2019). The consensus tree was visualized with FigTree v.1.4.4 (https://github.com/rambaut/figtree/releases).

Finally, for ease of comparison, a haplotype network was created by aligning *B. bairdii* , *B. arnuxii* and *B. minimus* mitochondrial control regions from this and previous studies (Dalebout et al. 2004; Kitamura et al. 2013; Morin et al. 2017; Yamada et al. 2019). Sequences were aligned in Geneious Prime 2020.1.2 with MAFFT v. 7.388 and the resulting alignment was pruned to the shortest control region fragment of 432 bp. A median‐joining network was created and visualized in PopART (Leigh and Bryant 2015) following the programme masking two indels at alignment positions 115 and 274. To infer the phylogenetic relationships of the control region sequences, the outgroup control region sequence from *M. europaeus* was aligned with the *Berardius* control region sequences using MAFFT, and a maximum likelihood tree was inferred with IQ‐tree and including 100 bootstrap replicates. The output tree was rooted to *M. europaeus* in Figtree.

## 3 Results

### 3.1 Five Novel *Berardius* Beaked Whale Genomes

Full nuclear genome data are lacking for many beaked whales, hindering detailed insights into their evolutionary history, as well as effective conservation and management measures. We generated five novel *Berardius* genomes at 13× and 21× depth of coverage for *B. minimus* , 6× and 32× depth of coverage for *B. bairdii* , and 27× depth of coverage for *B. arnuxii* . All samples resulted in mitogenomes with a depth of coverage greater than 100×.

### 3.2 Phylogenetic Analyses

To infer the evolutionary relationship and timing of *Berardius* speciation, we created separate calibrated Bayesian and maximum likelihood models for both the whole‐genome and mitogenome datasets. The species tree supported by the nuclear and mitochondrial datasets using both approaches supported *B. minimus* as being sister to *B. bairdii* plus *B. arnuxii* with Bayesian posterior values of 1 and maximum likelihood bootstrap support values of 100% at all species‐level nodes (Figures S1 and S2). Convergence of the Bayesian models was assessed in Tracer, with all parameters showing ESS values > 200.

**FIGURE 2:**
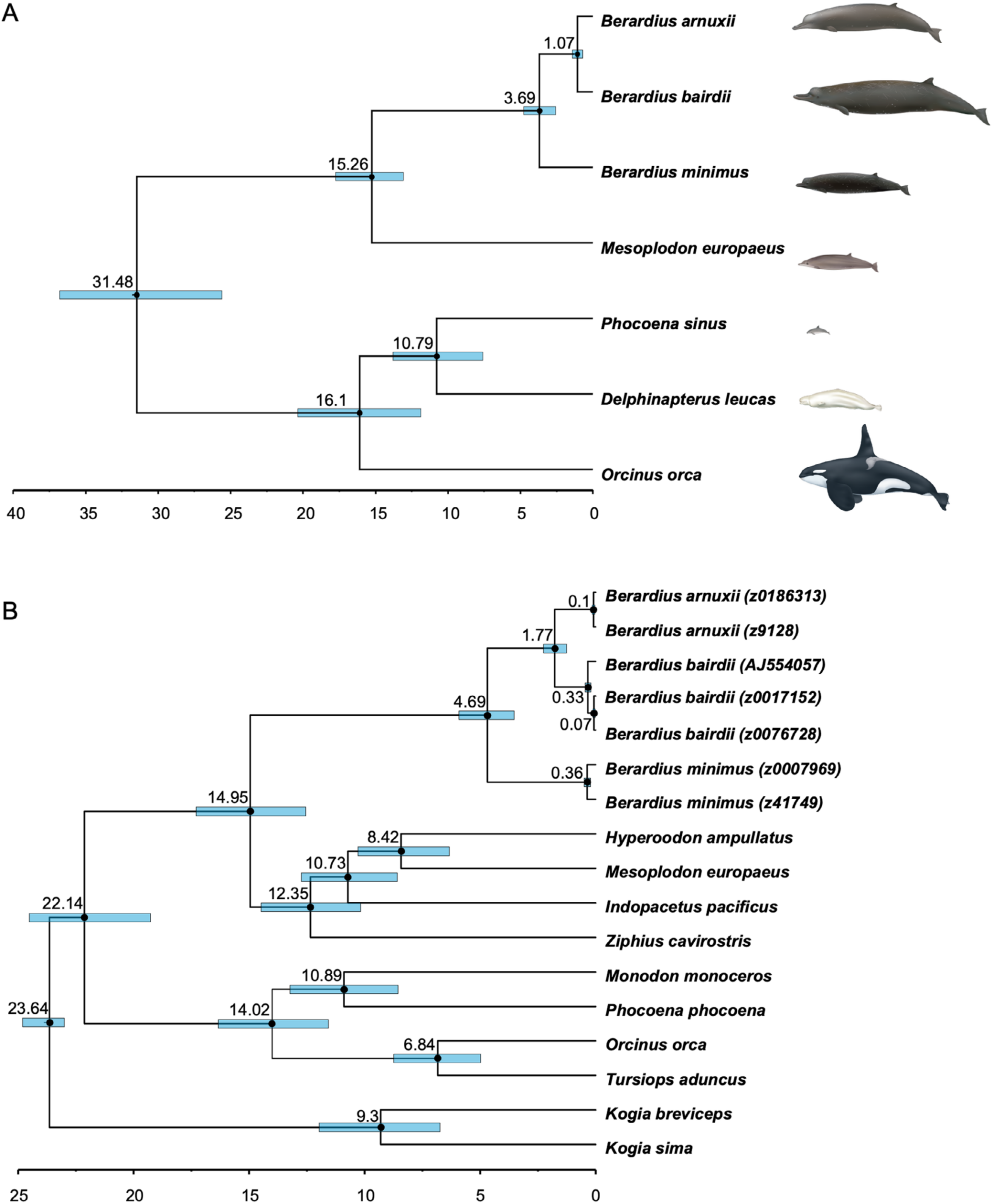
Evolutionary relationship and divergence among *Berardius* species and selected toothed whales. (A) Nuclear phylogeny and divergence times based on a 695,592 bp nuclear gene alignment. (B) Mitochondrial phylogeny and divergence times based on two rRNA and 13 protein‐coding genes of the mitogenomes included in Table S2. All node posterior support values were > 0.99. Blue bars represent 95% credible intervals and body sizes of cetacean illustrations are scaled according to maximum recorded body size as reported in Carwardine (2019).

The Bayesian nuclear genome phylogenetic analysis estimated a divergence time of ~3.69 (2.61–4.80 95% HPD) mya between *B. minimus* and the *B. bairdii* – *B. arnuxii* clade and ~1.07 (0.72–1.45 95% HPD) mya for the divergence of *B. bairdii* and *B. arnuxii* (Figure 2; Figure S3). The divergence time estimated in the Bayesian mitogenome phylogenetic tree for the split between *B. minimus* and the *B. bairdii* – *B. arnuxii* ancestor was ~4.69 mya (95% HPD: 3.53–5.93 mya), whereas *B. bairdii* and *B. arnuxii* diverged ~1.77 mya (95% HPD: 1.26–2.27 mya; Figure 2; Figure S4). To investigate the influence of gene flow on phylogenetic inferences, we assessed what proportion of the genome is in discordance with the species tree. Of the 2213 20 kb windows, we found 1500 (~68%) to follow the species tree (*M. europaeus*, (*B. minimus*, (*B. arnuxii*, *B. bairdii*))), and 441 (~20%) and 272 (~12%) to follow the alternative topologies (*M. europaeus*, (*B. arnuxii*, (*B. minimus*, *B. bairdii*))) and (*M. europaeus*, (*B. bairdii*, (*B. minimus*, *B. arnuxii*))), respectively. These results indicate nearly one‐third of the genome is in discordance with the species tree.

Finally, the haplotype network with the mitochondrial D‐loops sequenced in this study all fit within existing geographically mixed but species‐specific haplogroups (Morin et al. 2017) and were identical to haplotypes identified from samples sequenced in previous studies (Kitamura et al. 2013; Morin et al. 2017; Yamada et al. 2019; Figure S5). Similarly, the mitochondrial D‐loop maximum likelihood tree generally clustered sequences into clades by species, also supporting previous findings from these studies (Figure S5).

### 3.3 Gene Flow After Incipient Speciation

**FIGURE 3:**
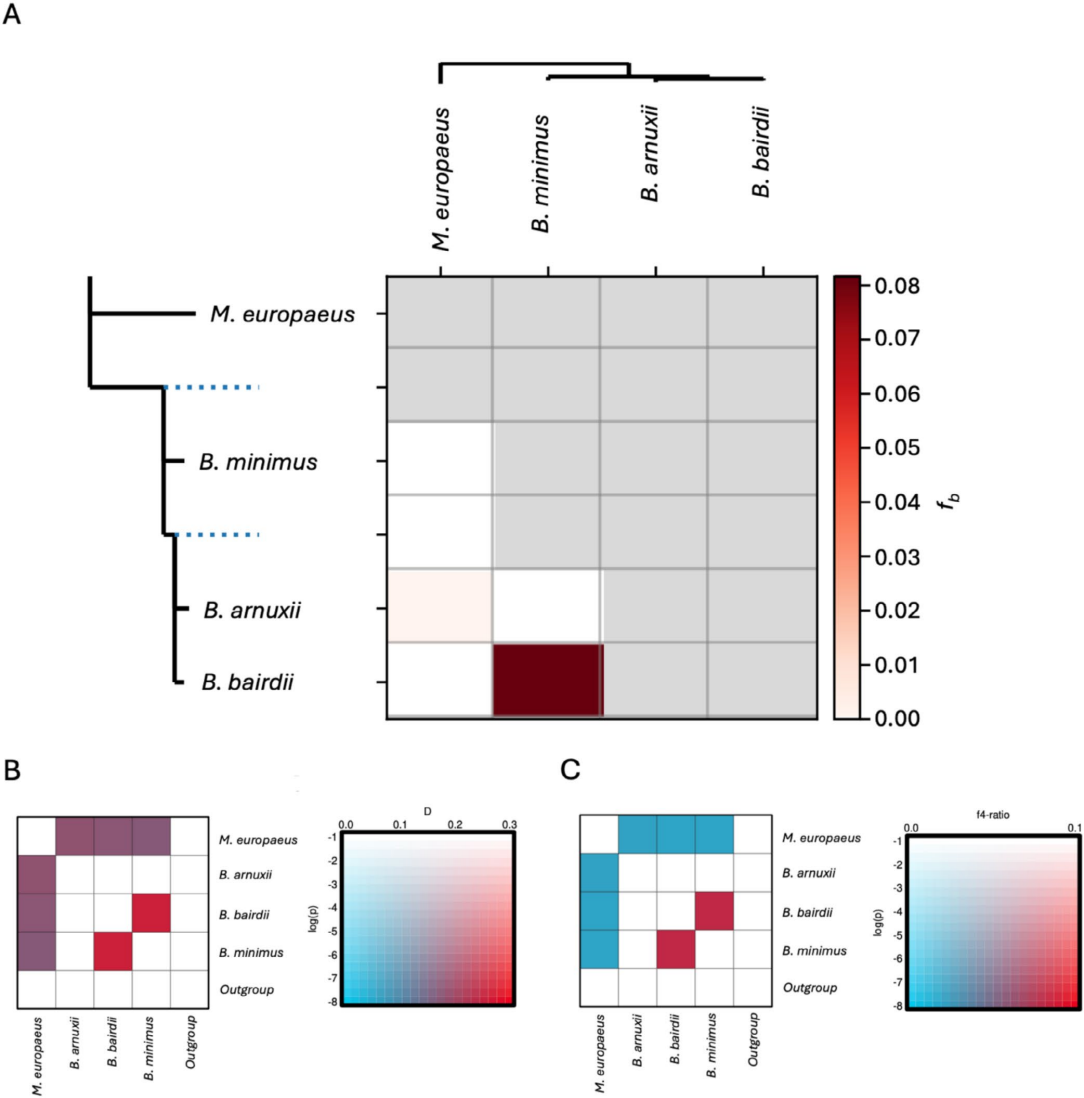
Gene flow visualizations within *Berardius* species using D‐suite for the (A) f‐branch statistic, (B) *D*‐statistic and (C) f4‐ratio statistic. The maroon and red coloured cells in the heatmaps show evidence for a strong hybridization signal between *B. bairdii* and *B. minimus* , the two *Berardius* species living in sympatry in the North Pacific.

We estimated the *D*‐statistic for the nuclear genome data to test for gene flow after the initial divergence between the three *Berardius* species. The analysis included *M. densirostris* as an outgroup and *B. minimus* , *B. bairdii* and *B. arnuxii* as ingroups. We found a much higher number of derived alleles shared between *B. bairdii* and *B. minimus* (457,672) than between *B. arnuxii* and *B. minimus* (269,077), which was supported by a *D*‐statistic of −0.26 and a significant |*Z*‐score| of 50.99, suggesting a higher rate of gene flow between *B. minimus* and *B. bairdii* (Table S5). This level of gene flow was supported by calculating the f4‐ratio (0.08) statistics using D‐suite, which suggests ~8% introgression between *B. minimus* and *B. bairdii* , but no detectable introgression involving *B. arnuxii* (Figure 3). The f‐branch analysis also indicated a low level (0.06%) of introgression between *B. arnuxii* and *M. europaeus* , which could indicate minimal historical gene flow but is more likely due to shared alleles resulting from incomplete lineage sorting relative to the outgroup species ( *Phocoena sinus* ) and to convergent base substitutions at some sites. Moreover, to estimate an interval for the last occurrence of gene flow between all *Berardius* species pairs, we employed the F1 hybrid PSMC (hPSMC) technique. The inferred intervals (recent‐ancient) were 300–800 kya for end gene flow between *B. minimus–B. bairdii*, 450–900 kya for end gene flow between *B. minimus* – *B. arnuxii* and 100–400 kya for *B. bairdii* – *B. arnuxii* (Table S6).

### 3.4 Genome‐Wide Genetic Distance Measurements

**FIGURE 4:**
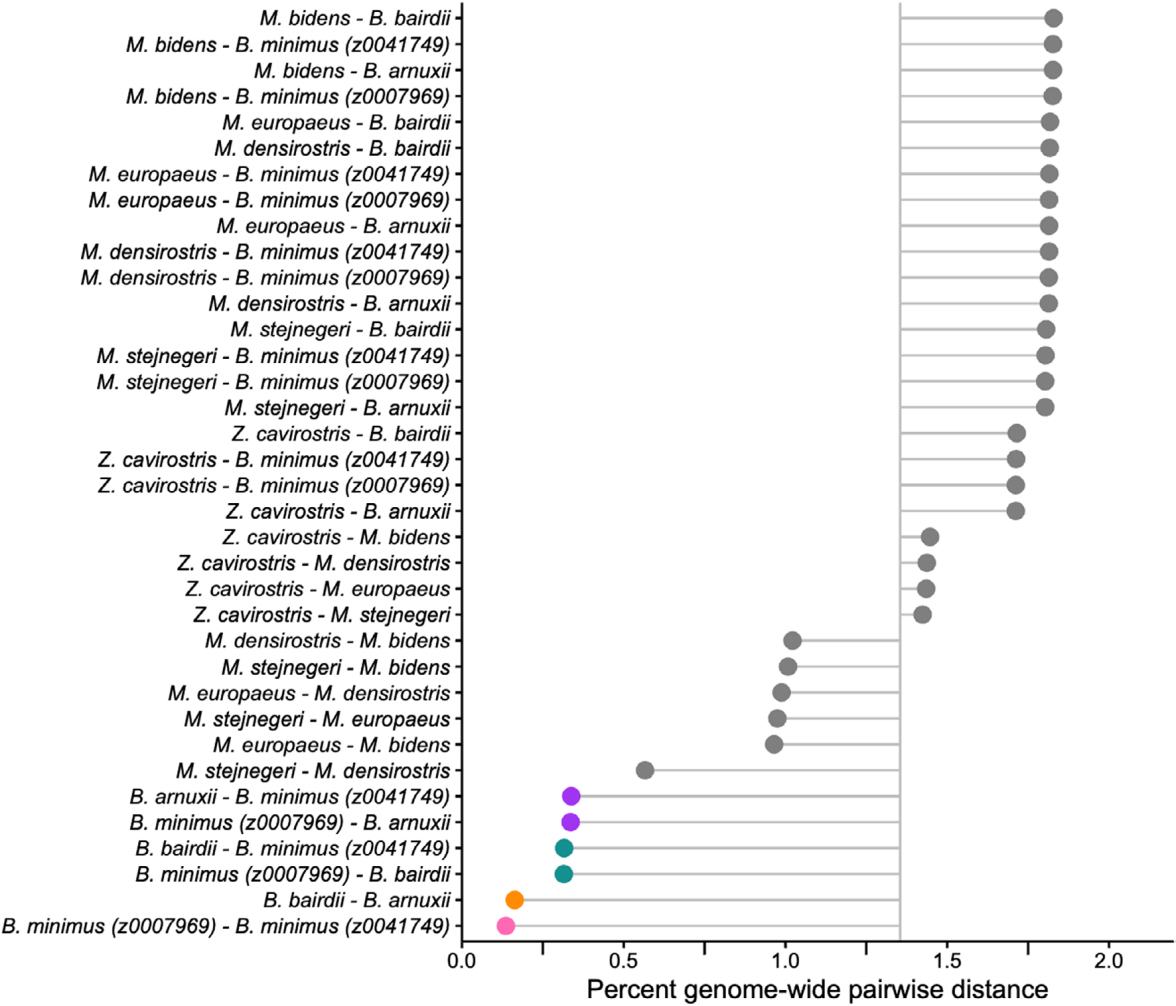
Genetic distance among *Berardius* and related beaked whales inferred using repeat masked autosomal percent pairwise distances comparisons (proportion differences/total sites) (Tables S1, S2 and S7). *B. bairdii* – *B. minimus* (cyan), *B. arnuxii* – *B. minimus* (purple) and *B. arnuxii* – *B. bairdii* (orange), *B. minimus–B. minimus* (pink). The vertical line represents the Ziphiidae average (1.4%).

**FIGURE 5:**
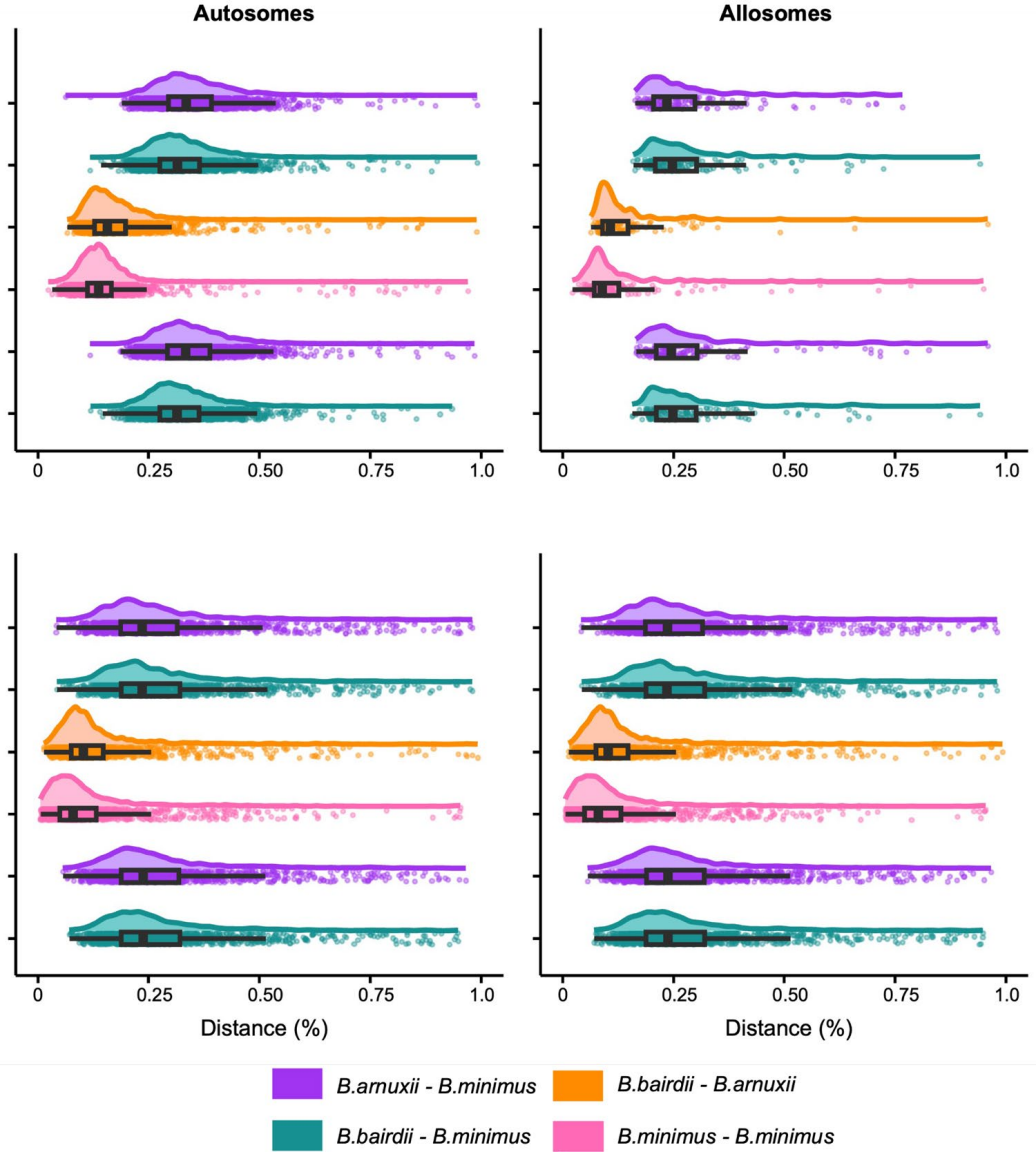
Genetic distance among *Berardius* beaked whales using non‐overlapping repeat masked autosomal (left) and allosomal (right) 1 Mb (top) and 100 kb (bottom) sliding window percent pairwise distances. The legend indicates two species included in a comparison. The top purple and cyan coloured raincloud plots in each panel include *B. minimus* sample z0041749, and the bottom two include *B. minimus* sample z0007969. Genetic distance restricted to < 1% for visualization. See Figure S7 for full distributions.

To further quantify the level of divergence between species pairs, we calculated pairwise genetic distances for the three *Berardius* species and included five additional beaked whale genomes to contextualize the results. The genome‐wide pairwise genetic distance was 0.16% between *B. arnuxii* and *B. bairdii* , 0.37% between *B. arnuxii* and *B. minimus* and 0.32% between *B. bairdii* and *B. minimus* (Figure 4; Table S7). All three species are genetically closer to each other than are all other beaked whale pairwise comparisons included in this study. To investigate whether there is an appreciable difference in genetic distances between the basal *B. minimus* and the two sister species *B. bairdii* and *B. arnuxii* , we looked at 1 Mb and 100 kb sliding windows across both allosomes (sex chromosomes) and autosomes (Figure 5; Figure S6). By comparing the distribution of distances for *B. minimus* – *B. bairdii* versus *B. minimus* – *B. arnuxii* , using the higher coverage *B. minimus* genome (z0041749), we found that allosomal distances were not significantly different for these species pairs but that autosomal distances were (allosomal 1 Mb and 100 kb; Kolmogorov–Smirnov *D* = 0.065, *p* > 0.902; *D* = 0.024, *p* > 0.799; autosomal 1 Mb and 100 kb Kolmogorov–Smirnov *D* = 0.125, *p* < 0.001; *D* = 0.078, *p* < 0.001; Table S8).

### 3.5 Demographic History and Heterozygosity Estimates

**FIGURE 6:**
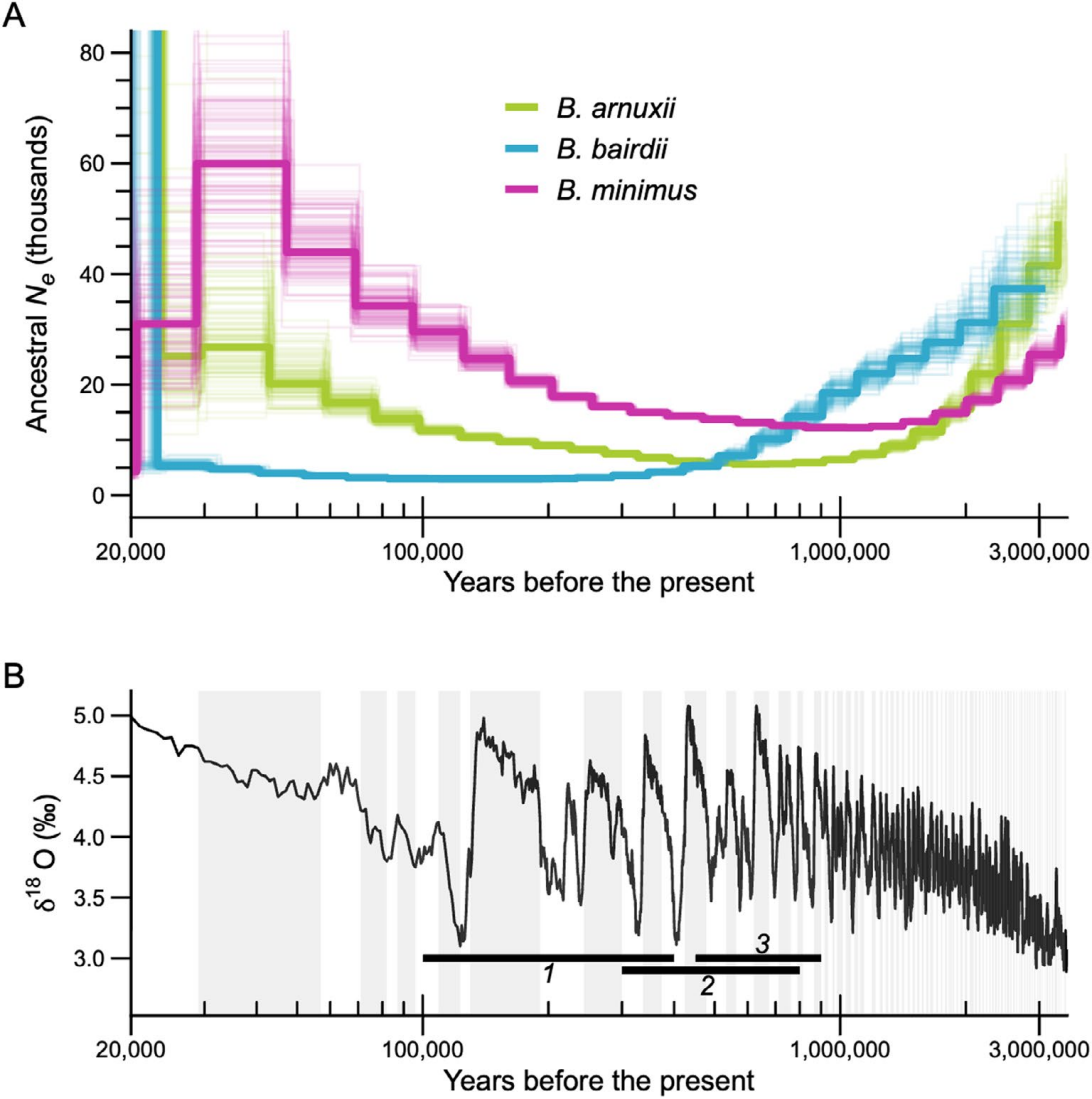
Demographic histories of *Berardius* beaked whales in relation to major climatic fluctuations. (A) PSMC demographic analysis for *B. minimus* (magenta), *B. bairdii* (blue) and *B. arnuxii* (green) scaled with a generalized generation time of 25.9 years and 1.15e‐8 substitutions/site/generation mutation rate. Past climate fluctuations inferred using oxygen isotope date from 57 stacked sediment cores (Lisiecki and Raymo 2005) with shaded marine isotope stages. The hPSMC end of gene flow intervals for (1) *B. bairdii–B. arnuxii*, (2) *B. minimus–B. bairdii* and (3) *B. minimus–B. arnuxii* are overlayed as horizontal lines on the oxygen isotope readings. Shaded/unshaded bands represent marine isotope transitions (Lisiecki and Raymo 2005). Both A and B are logarithmic scales and represent years before present.

We used PSMC analyses to infer the historical demography of all three species (selecting the higher coverage *B. minimus* genome) up to the Last Glacial Maximum (LGM; 21 kya) (Figure 6). All three species showed a gradual decline in effective population size (*N*_e_) leading up to the Mid‐Pleistocene Transition (MPT; 1.3–0.7 Ma). Following the MPT, the *N*_e_ of *B. minimus* and *B. arnuxii* steadily increased, reaching a maximum at ~30 kya, after which *B. minimus* experienced a sharp decline. *B. bairdii* continued to decline following the MPT until 300 kya, when *N*_e_ stabilized at low *N*_e_ leading up to the onset of the LGM. Between 30 and 20 kya, *B. arnuxii* and *B. bairdii* experienced a drastic increase in *N*_e_, accompanied by wide bootstrap confidence intervals. This recent increase should be interpreted with caution, as PSMC is less reliable at more recent time periods (Schiffels and Durbin 2014; Terhorst et al. 2017).

**FIGURE 7:**
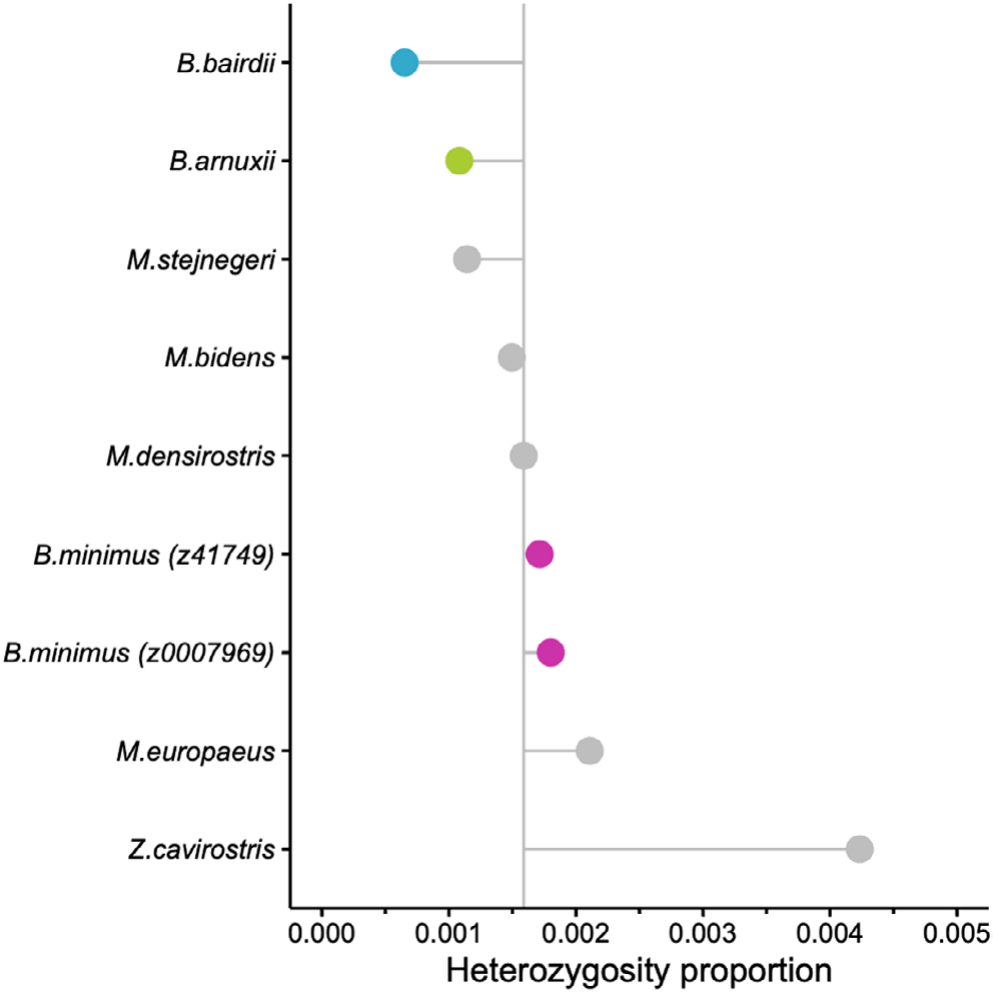
Genetic diversity inferred using repeat masked genome‐wide autosomal heterozygosity estimates (proportion heterozygous sites/total sites) using strict filtering of sites to limit the impact of DNA damage on heterozygosity estimates with *B. minimus* (magenta), *B. bairdii* (blue) and *B. arnuxii* (green). The vertical line represents the median heterozygosity (0.0016) for all species included in the comparison.

To further investigate the large *B. minimus* *N*_e_ and inform the speciation process of *Berardius* beaked whales, we calculated genome‐wide heterozygosity for all three *Berardius* species and for five additional beaked whale species. We found that *B. minimus* had the highest repeat masked genome‐wide heterozygosity of all *Berardius* beaked whales (Figure 7). In all beaked whale heterozygosity calculations, masking of repetitive regions reduced average heterozygosity by ~7% relative to unmasked genomes (Table S9). Visualizing the distribution of heterozygous base calls relative to the alternative allele frequency (Figure S7) showed high levels of heterozygous calls at low frequencies (i.e., < 0.25) but with similar patterns between individuals to the exclusion of *B. minimus* (z0007969). *B. minimus* (z0007969) had clearly elevated levels of heterozygous base calls at lower alternative allele frequencies. *B. arnuxii* had a higher level of heterozygosity than *B. bairdii* , a result consistent with the higher long‐term *N*_e_ reported in the PSMC analysis.

## 4 Discussion

### 4.1 Genomic Data Are Consistent With Three *Berardius* Species

The presence of two types of *Berardius* in the North Pacific was suggested as early as 1955 by Omura et al. (1955) on the basis of reports from whalers. Kitamura et al. (2013), however, provided the first molecular evidence to suggest the separation of *Berardius* into three forms and Morin et al. (2017) expanded upon mitochondrial control region (mtDNA CR) data and found pairwise comparisons of the three *Berardius* clades to be characteristic of divergence at the species level. However, it was not until Yamada et al. (2019) that external skull morphology and mtDNA CR data were synthesized, and the new species designation of *B. minimus* , Sato's beaked whale, was proposed. Our genome‐wide pairwise genetic distances (Figure 4), deep phylogenomic divergence estimates and high node posterior probabilities in both the nuclear and mitochondrial genomes (Figure 2) support these previous findings. While there is no quantitative framework guiding species designations based on genome pairwise distances, the average autosomal genetic distances between *B. minimus* – *B. bairdii* (0.32%) and *B. minimus* – *B. arnuxii* (0.34%) are greater than between another recently described beaked whale species pair; True's and Ramari's beaked whales (*M. mirus–M. eueu*: 0.28%; Carroll et al. 2021) supporting the designation of *B. minimus* as a separate species. Intriguingly, the antitropically distributed and well‐recognized sister species ( *B. bairdii* and *B. arnuxii* ) have the lowest pairwise distances (0.16%) known among beaked whales compared in this study, suggesting equatorial thermal barriers to gene flow may be a relatively recent driver of species separation in these two species.

### 4.2 North Pacific Origin and Divergence of *Berardius*

The nuclear and mitochondrial genome phylogenies presented here both complement and improve upon previous phylogenetic reconstructions of the genus *Berardius* based on short DNA sequence fragments (Kitamura et al. 2013; Morin et al. 2017; Yamada et al. 2019). We found high posterior probabilities supporting a phylogenetic tree in which the three *Berardius* species originated through an initial divergence between *B. minimus* with the ancestor of *B. bairdii* and *B. arnuxii* , with a more recent split between *B. bairdii* and *B. arnuxii* . This points to a North Pacific origin and early diversification of *Berardius* within this ocean basin, followed by colonization of the southern Hemisphere by *B. arnuxii* or its ancestor. Fossil information from extinct species that are closely related to extant *Berardius* is sparse (Bianucci et al. 2024), but a North Pacific origin of *Berardius* agrees well with the *Berardius kobayashii* from the western North Pacific from the Middle to Late Miocene (Kawatani and Kohno 2021). It cannot be ruled out that *Berardius* originated in the southern Hemisphere and subsequently colonized the North Pacific (Morin et al. 2017). However, this is the least parsimonious explanation, implying two trans‐equatorial dispersal events—first by *B. minimus* and later by *B. bairdii—*and is not supported by the current genomic and fossil data. Another alternative hypothesis is that under different cooler climatic conditions in the deep past, *Berardius* may have had a broader distribution and vicariance within *Berardius* occurred upon warming of the climate. Future discovery and dating of *Berardius* fossils could help to refine the evolutionary origin of the genus.

The divergence time estimates derived from the calibrated nuclear and mitochondrial genome phylogenies are in overall agreement, pointing to a split between *B. minimus* and the *B. bairdii* – *B. arnuxii* ancestor approximately 5–4 mya and a split between *B. bairdii* and *B. arnuxii* approximately 2–1 mya. The latter fits well within the range of a previous mitogenome and nuclear gene‐based estimate of 2.9 mya (95% HPD: 0.68–5.81; McGowen et al. 2009). The timeframe for speciation within *Berardius* beaked whales spans a period of major climatic fluctuations, with the divergence of *B. minimus* overlapping with the Miocene–Pliocene transition (~5 Ma), and the split between *B. bairdii* and *B. arnuxii* following the super‐interglacial MIS 31 (1.085–1.055 mya; Lisiecki and Raymo 2005) and following the Pliocene‐Pleistocene transition (2.5 Ma) (Zijderveld et al. 1986; Lisiecki and Raymo 2007). The period of *Berardius* speciation also saw several palaeoceanic events (e.g., closure of the Indo Pacific Seaway and Panama Seaway), and was characterized by changing thermohaline circulation, upwelling patterns and global marine productivity (Peterson et al. 1992; Srinivasan and Sinha 1998; Steeman et al. 2009; Zhang et al. 2017).

We hypothesize a prominent role of these climatic and oceanic events in the speciation of *Berardius* beaked whales and propose that a change in ecological niche within the present‐day North Pacific initiated a divergence between the relatively small *B. minimus* and the presumably larger ancestor of *B. bairdii* and *B. arnuxii* . Indeed, one study comparing stable isotopes, albeit limited in sample size, reported evidence for ecological niche partitioning between *B. minimus* and *B. bairdii* based on differences in *δ* ^13^C and THg concentrations, suggesting a more bathypelagic foraging habitat for *B. bairdii* (Endo et al. 2024). *B. bairdii* has a maximum recorded dive of 1777 m (Minamikawa et al. 2007) and stomach content analysis showed that they feed on squid and demersal (bottom‐dwelling) fish at depths of at least 1000 m (Ohizumi et al. 2003), suggesting that *B. bairdii* is capable of and regularly undertakes deep sea foraging dives. Collecting dive profiles and stomach contents for *B. minimus* should be future research priorities to further investigate this ecological niche partitioning hypothesis. In odontocetes, a positively significant correlation has been observed between body size and dive depth (Noren and Williams 2000), and it has been proposed that deep diving removes competition between sympatric species, rewarding deep divers with access to larger, more nutrient‐rich prey (Visser et al. 2021). Along these lines, we hypothesize that a larger body size in the ancestor of *B. bairdii* and *B. arnuxii* may have allowed it to access a novel prey base and gradually resulted in its ecological specialization and speciation, as observed in other marine mammals (Riesch et al. 2012; Ansmann et al. 2015; Moura et al. 2015; Foote et al. 2016; Costa et al. 2021). Indeed, it has been hypothesized that toothed whales evolved gigantism because a larger body size allowed for the progressive invasion of the deep sea (Goldbogen and Madsen 2018). The extinct *B. kobayashii* was ~50% smaller than *B. minimus* (Kawatani and Kohno 2021), indicating that an increase in body size within *Berardius* predates the full transition to gigantism in *B. bairdii* and *B. arnuxii* .

A long‐standing hypothesis for the formation of antitropically distributed sister species is that populations of temperate species are pushed towards lower latitudes during cooler global conditions and are separated by the subsequent formation of thermal gradients (White 1986). This results in a North–South disjunct distribution with an equatorial thermal barrier dividing populations and species, which has been suggested to be the allopatric speciation process for *B. bairdii* – *B. arnuxii* (Steeman et al. 2009; Fordyce 2018). Our results suggest the *B. bairdii* – *B. arnuxii* speciation process was more complex than a single bifurcation, with at least one period of secondary contact and gene flow after initial divergence. Still, our divergence estimates overlap well with the super‐interglacial period MIS 31 (1.08–1.06 mya) during the MPT, indicating that a thermal barrier was likely involved in the speciation between *B. bairdii* and *B. arnuxii* . MIS 31 is considered to be a particularly warm and extended super‐interglacial characterized by high sea‐surface temperatures, a shift poleward of the meridional thermal gradient in the Northern Hemisphere and a decrease in sea surface ice (Froelich et al. 1991; Scherer et al. 2003; Maiorano et al. 2009; Justino et al. 2017; Beltran et al. 2020). Thus, it is plausible that the super‐interglacial MIS 31 could have played a role in initially separating ancestral populations either allopatrically or parapatrically.

### 4.3 Speciation in the Presence of Gene‐Flow

The inferred cessation of gene flow between *B. minimus* and *B. arnuxii* (0.45–0.90 mya) broadly overlaps with that between *B. minimus* and *B. bairdii* (0.30–0.80 mya) using the hPSMC method (Figure 6). This overlap precludes a definitive temporal ordering of the two divergence events; however, the estimates are consistent with gene flow persisting somewhat longer between *B. minimus* and *B. bairdii* than between *B. minimus* and *B. arnuxii* . This pattern is compatible with *B. minimus* and *B*. *bairdii* being sympatric and thereby creating opportunities to interbreed more easily than *B. minimus* with *B. arnuxii* . Ultimately, the end of gene flow between *B. minimus* and *B. bairdii* may be explained by an ecological specialization driving a divergence between the smaller *B. minimus* and larger *B. bairdii* species, or alternatively, the passage of enough evolutionary time and accumulated genetic changes may have prevented viable offspring at some stage. Despite their current disjoint antitropical distribution, we found that gene flow between *B. bairdii* and *B. arnuxii* occurred as recently as 0.10–0.40 mya, overlapping with the unusually cold MIS6 glacial period (0.191–0.130 mya; Lisiecki and Raymo 2007) and followed by the onset of the warm Eemian interglacial (0.126 mya; NEEM Community Members 2013). The hPSMC method does not detect the number of introgression events, but we hypothesize that since their initial divergence at the Pliocene–Pleistocene transition, glacial periods may have pushed both species towards equatorial latitudes where they interbred and introgression occurred, whereas warmer interglacials separated the two species at temperate latitudes. Although *B. bairdii* and *B. arnuxii* are thought to mainly inhabit temperate to subpolar ranges, scarring from encounters with tropical‐subtropical cookie cutter sharks ( *Isistius brasiliensis* ) (Dwyer 2011) suggests possible migrations into warmer waters. Indeed, *B. bairdii* has previously been documented to migrate in response to changes in sea surface temperature, with animals shifting between offshore habitats during winter and spring and the continental slope during summer when water temperatures increase (Dohl 1983). Periodic range overlaps during migrations and hence opportunities for gene flow may have persisted at the edges of the lower latitude ranges until the two species became completely isolated by an equatorial warm water boundary. Interestingly, another antitropically distributed pair of ziphiid sister species, True's beaked whale ( *Mesoplodon mirus* ) in the North Atlantic and Ramari's beaked whale (*Mesoplodon eueu*) in the Southern Ocean, also were inferred to have had a relatively recent cessation of gene flow 0.35–0.55 mya (Carroll et al. 2021). These shared north–south patterns suggest that relatively recent global climatic and/or oceanographic event(s) were a common force in the antitropical speciation of these beaked whales and possibly other marine vertebrates.

The process of speciation in marine environments is poorly understood due to inadequately characterized seascapes, insufficient resources for conducting marine surveys, the challenge of quantifying reproductive isolation/barriers in situ, and a general lack of biogeographical classifications for vast areas of the ocean (Faria et al. 2021). We demonstrate that speciation in *Berardius* was more complex than a single barrier forming between two species, with gene flow persisting for several million years after initial divergence, corresponding to at least 75% of the post‐divergence branch length between all *Berardius* species pairs. Similar patterns of gene flow have been observed between odontocetes within the superfamily Delphinoidea (Westbury et al. 2021; Moura et al. 2020 within Delphinidae) and also in Mysticeti (Árnason et al. 2018; Springer et al. 2020; Wolf et al. 2023). Thus, evidence is accumulating that marine speciation is a complex process involving periods of introgression and divergence, illustrated by the three *Berardius* species, which appear to be the result of alternating periods of sympatric, allopatric and parapatric diversification, as well as prolonged episodes of gene flow between sister species but also more distantly related species pairs.

### 4.4 Demographic History

The demographic history analysis suggests that *Berardius* species populations have generally declined during periods of global cooling, or that populations became isolated from one another, leading to decreased connectivity. Cooler conditions could have resulted in reduced suitable habitat and contracted species' ranges to lower latitudes, though past environmental reconstructions should be investigated to test this hypothesis. Although the Pleistocene (beginning ~2.5 mya) experienced many cycles of ice ages and interglacials, the global temperature trend was decreasing, which corresponds to a decline or flattening in effective population size for *Berardius* species until 0.300–0.600 mya. While *B. bairdii* maintained a low population size leading up to the Last Glacial Maximum (LGM; 0.021 mya), the effective population size for *B. minimus* and *B. arnuxii* began to increase, especially following the markedly warm Eemian interglacial (~126 kya). The increase in population size for both continued until just before the LGM (21 kya), when it declined for *B. minimus* . Indeed, there is a potential for false increases in *N*_e_ in recent time intervals using the method applied here that are due to population subdivision or gene flow (Hilgers et al. 2025); therefore the decline in *N*_e_ for all three species should be interpreted with caution until substructure across their respective ranges is further investigated. It is surprising however, that leading up to the LGM, *B. minimus* had the largest effective population size of all three *Berardius* species since it appears to be the least abundant and most geographically restricted in the present, but lacks any indication of reduced heterozygosity or gene flow with *B. bairdii* or *B. arnuxii* for at least 0.300 mya. It is possible that *B. minimus* is more abundant than currently assumed or that its high levels of heterozygosity are the result of relatively older introgression with *B. bairdii*, and/or population structure within *B. minimus* . The larger *N*_e_ and greater heterozygosity estimate of *B. arnuxii* compared to *B. bairdii* may be driven by the larger suitable habitat of the Southern Ocean habitat of the former, compared to the smaller North Pacific range of the latter.

## 5 Conclusion and Perspectives

By generating high‐coverage mitochondrial and nuclear genomes, we provide valuable genomic resources for the mysterious and understudied beaked whale family. We document the speciation process within *Berardius* and confirm *B. minimus* as sister to a *B. bairdii* + *B. arnuxii* clade with divergences dated to the Plio‐Plestocene. We hypothesize that speciation was driven by resource specialization and an increase in body size in the common ancestor of *B. bairdii* and *B. arnuxii* . We detected continued gene flow patterns after incipient speciation in the Late Miocene, with a prominent signal for the sympatric species *B. minimus* and *B. bairdii* . We further hypothesize that warming during the Eemian interglacial was a secondary driver of speciation, causing the ultimate cessation of gene flow for the presently antitropically distributed *B. bairdii* and *B. arnuxii* . Our demographic inference can be used to inform species knowledge on long‐term population sizes and how past climate has influenced species population trajectories. The use of beach‐cast samples demonstrates the capacity of genomics to assist in the study of otherwise cryptic and hard to access species like beaked whales.

## Author Contributions

M.L.M., M.T.O. and P.A.M. conceived and designed the study; P.A.M. provided materials and analysed data; M.S.S., J.G. and M.T.O. provided funding and data; M.L.M. and M.V.W. analysed the data; M.L.M. drafted the manuscript; M.L.M., M.T.O., M.V.W., J.G. and P.A.M. edited the manuscript; all authors approved the final version.

## Funding

This work was supported by Horizon 2020 Research and Innovation Programme (801199), Carlsbergfondet (CF21‐0425), National Science Foundation (1457735) and SWFSC Marine Mammal and Turtle Division.

## Conflicts of Interest

The authors declare no conflicts of interest.

## Supporting information


**Figure S1:** Maximum likelihood tree based on a 695,592 bp nuclear gene alignment. Internal nodes are labelled with the bootstrap support percentage out of 10,000 replicates. Branch lengths represent the number of substitutions per site, with the scale bar acting as a point of reference.
**Figure S2:** Maximum likelihood mitogenome phylogeny. Internal nodes are labelled with the bootstrap support percentage out of 10,000 replicates. Branch lengths represent the number of substitutions per site, with the scale bar acting as a point of reference.
**Figure S3:** MCMCtree Bayesian calibrated tree based on a 695,592 bp nuclear gene alignment. Internal nodes are labelled with mean divergence values and 95% credible intervals. Ages are presented in millions of years ago.
**Figure S4:** Bayesian time‐calibrated mitogenome phylogeny. Node labels correspond to mean divergence time and 95% HPD credible intervals. Ages are presented in millions of years ago.
**Figure S5:** (A) Median‐joining network of 35 D‐Loop sequences representative of *B. bairdii* , *B. arnuxii* and *B. minimus* haplotypes. Newly assembled mtDNA by this study are coloured red. Hash marks represent single fixed differences between haplotypes. (B) Maximum likelihood tree of the 35 D‐loop sequences rooted with *M. europaeus* as an outgroup.
**Figure S6:** Genetic distance among *Berardius* beaked whales using non‐overlapping repeat masked autosomal (left) and allosomal (right) 1 Mb (top) and 100 kb (bottom) sliding window percent pairwise distances. The legend indicates two species included in a comparison. The top purple and cyan coloured raincloud plots in each panel include *B. minimus* sample z0041749, and the bottom two include *B. minimus* sample z0007969.
**Figure S7:** Counts of alternative alleles separated based on their alternative allele frequencies showing the lower coverage *B. minimus* sample z0007969 had clearly elevated levels of heterozygous base calls at lower alternative allele frequencies.

**Table S1:** Sample information and mapping results for *Berardius* beaked whales sequenced in this study.
**Table S2:** Information on the genome assemblies and/or raw read data accessed for nuclear analyses as well as input parameters for the nuclear phylogeny in MCMCtree.
**Table S3:** Mitogenome accessions or raw data used to assemble mitogenomes for the mitochondrial phylogeny in Figure 3.
**Table S4:** Partitions and nucleotide substitution models used for the mitochondrial genome calibrated phylogeny in BEAST.
**Table S5:** Results from the ABBA‐BABA tests. The test follows (((P1, P2), P3)O), where P1, P2 and P3 are the species of interest and O is an outgroup (not included in the table). A theoretical basis behind the test is that without introgression, the allelic patterns for ancestral (A) and derived (B) alleles across the four genomes should be equally split between ABBA or BABA patters (i.e., P2 should share an equal number of derived alleles with P3 as it does with P1), resulting in a *D*‐statistic of zero. An excess in ABBA patterns suggests introgression between P2 and P3 (positive *D*‐statistic), whereas an excess in BABA patterns suggests introgression between P1 and P3 (negative *D*‐statistic).
**Table S6:** Inferred lower (recent) and upper (ancient) bounds for estimating end of gene flow for *Berardius* lineage pairings and the pre‐divergence effective population size used as input for simulations.
**Table S7:** Genome‐wide autosomal pairwise percent distances between *Berardius* species and select beaked whale outgroup species.
**Table S8:** Results from Kolmogorov–Smirnov tests calculating whether the distribution of distance across 1 Mb and 100 kb autosomal and allosomal windows between species is significant. Significant tests with a *p*‐value < 0.05 have an * symbol.
**Table S9:** Proportion heterozygous sites calculated with autosomes, repeat masked autosomes and after filtering sites to account for DNA damage or sequencing errors following Hempel et al. (2024)*, for Berardius beaked whales and select beaked whale outgroups.

## Data Availability

All data accessible under the BioProject PRJNA521413. Scripts used in the analyses are available on GitHub: https://github.com/Morgan‐McCarthy/Berardius_phylogenomics.
