# Supplementary material for: Genomic Insights Into the Evolutionary History of Berardius Beaked Whales: Speciation Driven by Resource Specialization, Gigantism and Thermal Barriers?
Source: Mol Ecol. 2026 Jun 17;35(12):e70426. doi: 10.1111/mec.70426 (PMC13275336; doi:10.1111/mec.70426)
Supplement: Supplementary file 1 — Figure S1: Maximum likelihood tree based on a 695,592 bp nuclear gene alignment. Internal nodes are labelled with the bootstrap support percentage out of 10,000 replicates. Branch lengths represent the number of substitutions per site, with the scale bar acting as a point of reference. Figure S2: Maximum likelihood mitogenome phylogeny. Internal nodes are labelled with the bootstrap support percentage out of 10,000 replicates. Branch lengths represent the number of substitutions per site, with the scale bar acting as a point of reference. Figure S3: MCMCtree Bayesian calibrated tree based on a 695,592 bp nuclear gene alignment. Internal nodes are labelled with mean divergence values and 95% credible intervals. Ages are presented in millions of years ago. Figure S4: Bayesian time‐calibrated mitogenome phylogeny. Node labels correspond to mean divergence time and 95% HPD credible intervals. Ages are presented in millions of years ago. Figure S5: (A) Median‐joining network of 35 D‐Loop sequences representative of B. bairdii , B. arnuxii and B. minimus haplotypes. Newly assembled mtDNA by this study are coloured red. Hash marks represent single fixed differences between haplotypes. (B) Maximum likelihood tree of the 35 D‐loop sequences rooted with M. europaeus as an outgroup. Figure S6: Genetic distance among Berardius beaked whales using non‐overlapping repeat masked autosomal (left) and allosomal (right) 1 Mb (top) and 100 kb (bottom) sliding window percent pairwise distances. The legend indicates two species included in a comparison. The top purple and cyan coloured raincloud plots in each panel include B. minimus sample z0041749, and the bottom two include B. minimus sample z0007969. Figure S7: Counts of alternative alleles separated based on their alternative allele frequencies showing the lower coverage B. minimus sample z0007969 had clearly elevated levels of heterozygous base calls at lower alternative allele frequencies. [file MEC-35-e70426-s001.docx]

Supplementary Figures


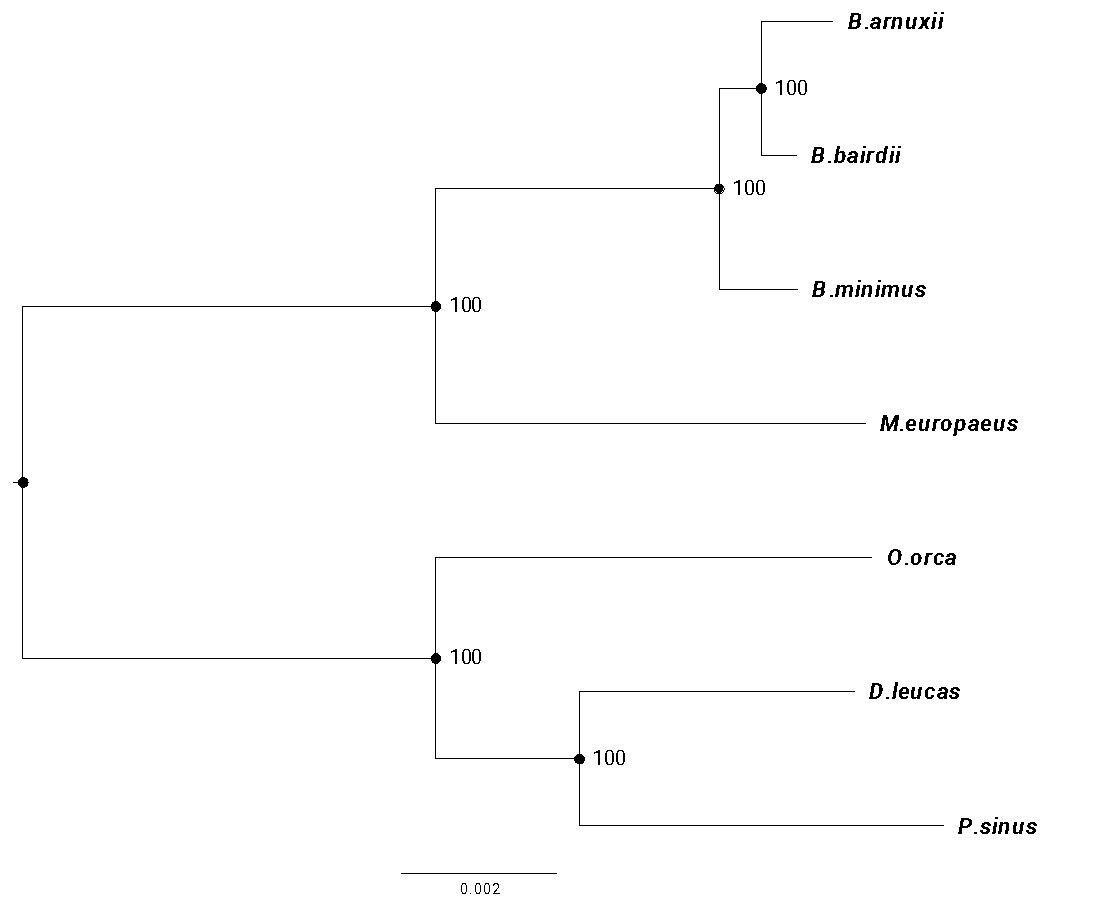


Supplementary Figure S1: Maximum likelihood tree based on a 695,592 bp nuclear gene alignment. Internal nodes are labeled with the bootstrap support percentage out of 10,000 replicates. Branch lengths represent the number of substitutions per site, with the scale bar acting as a point of reference.


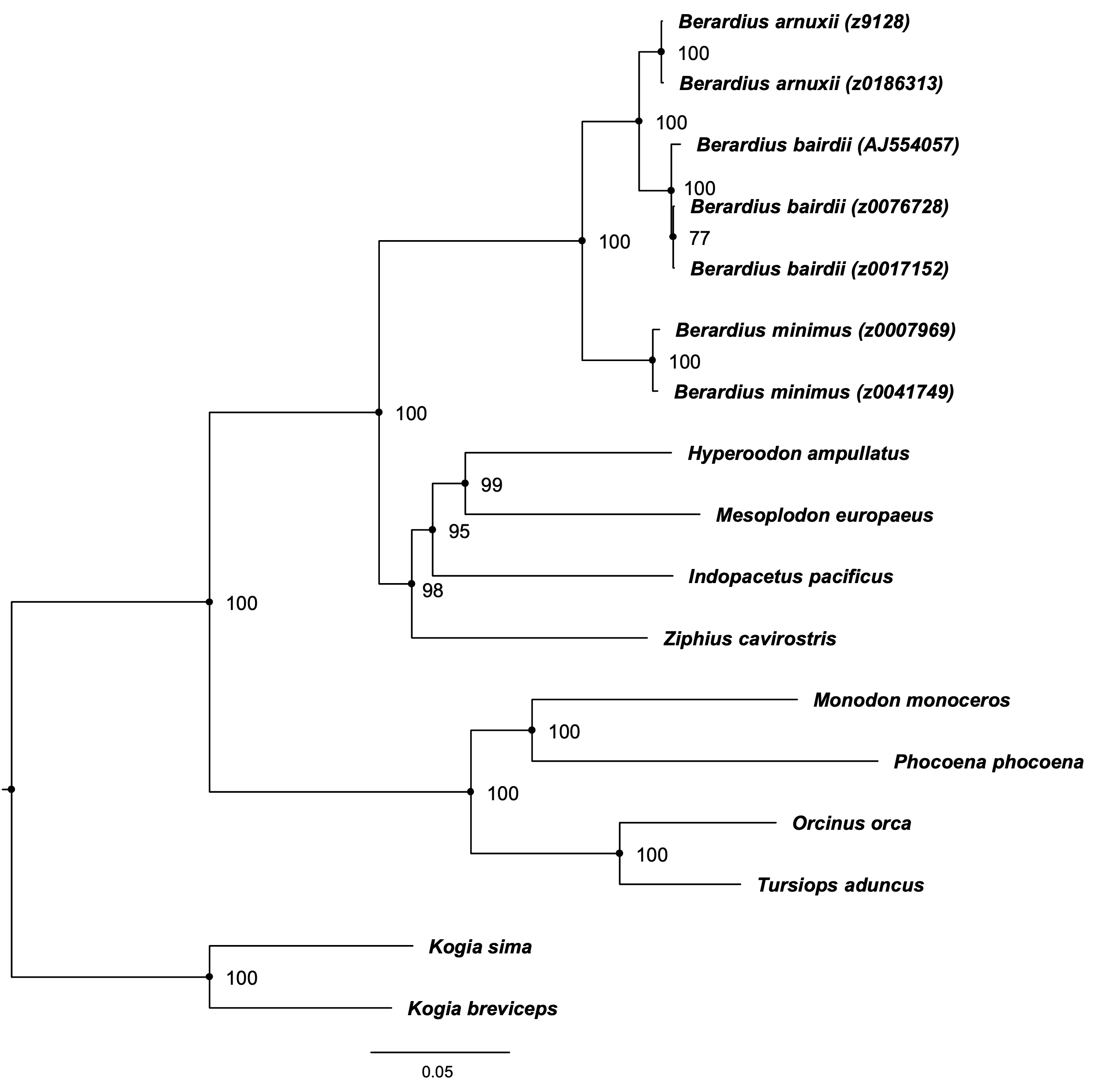


Supplementary Figure S2: Maximum likelihood mitogenome phylogeny. Internal nodes are labeled with the bootstrap support percentage out of 10,000 replicates. Branch lengths represent the number of substitutions per site, with the scale bar acting as a point of reference.


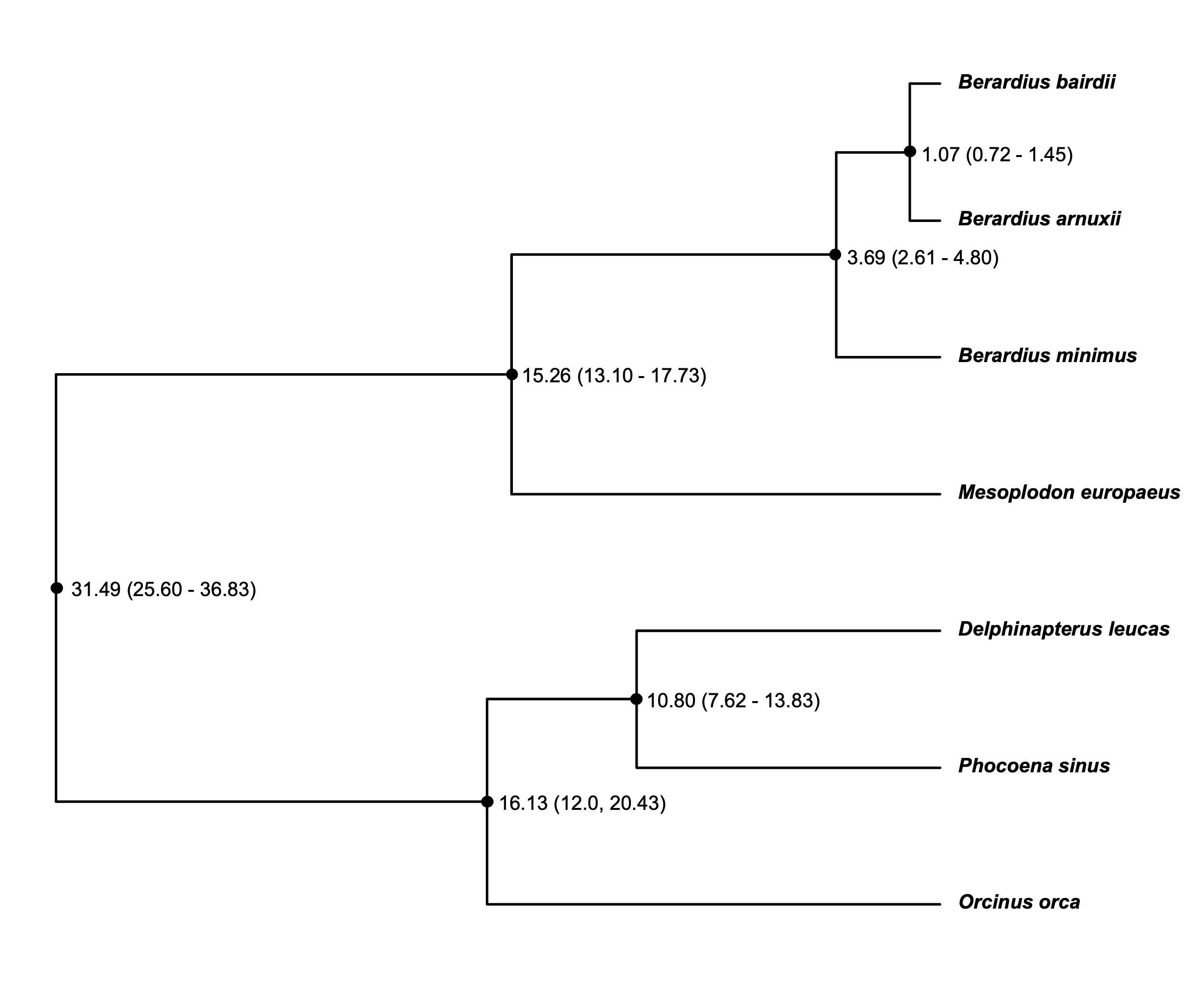


Supplementary Figure S3: MCMCtree Bayesian calibrated tree based on a 695,592 bp nuclear gene alignment. Internal nodes are labeled with mean divergence values and 95% credible intervals. Ages are presented in millions of years ago.


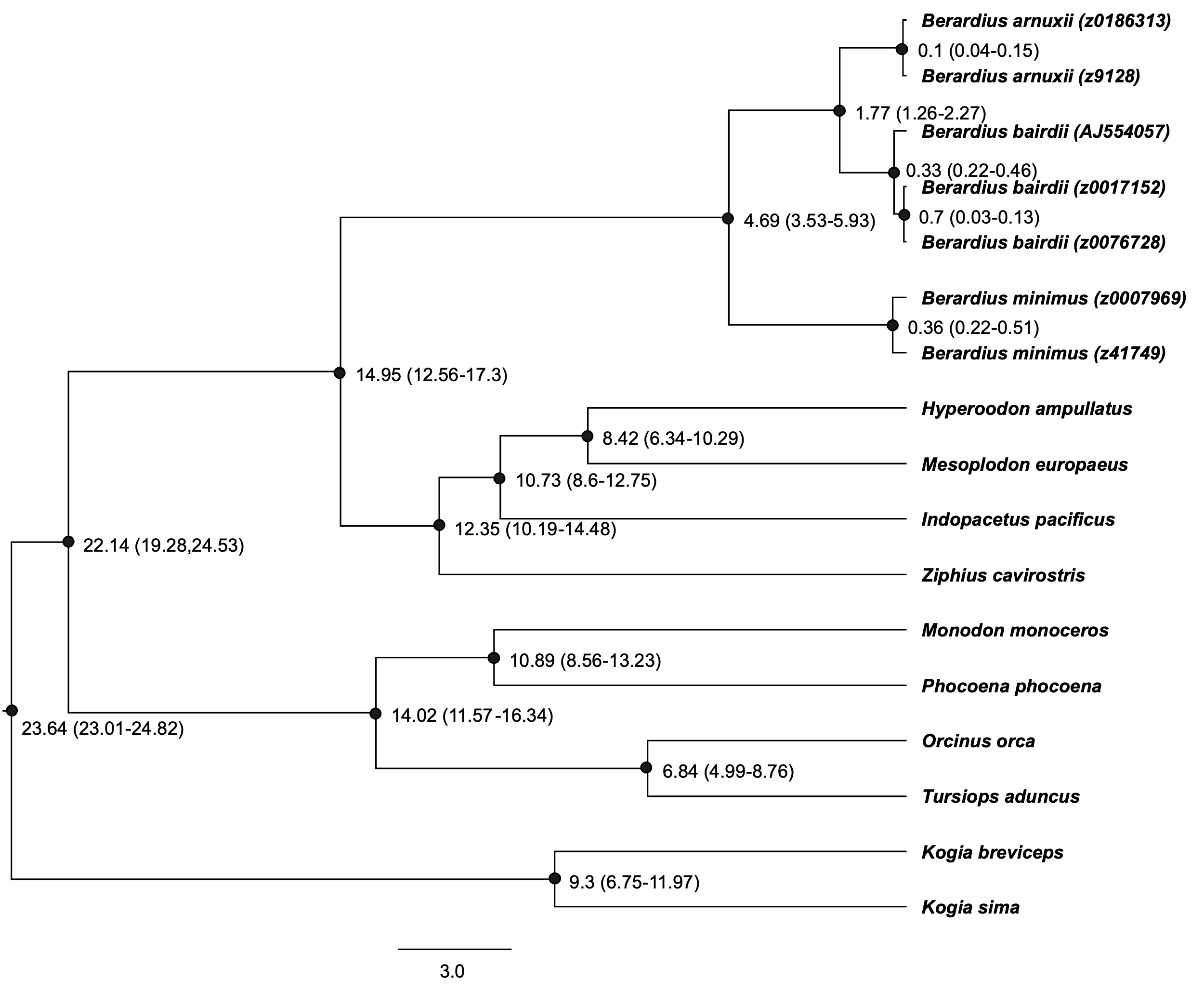


Supplementary Figure S4: Bayesian time-calibrated mitogenome phylogeny. Node labels correspond to mean divergence time and 95% HPD credible intervals. Ages are presented in millions of years ago.


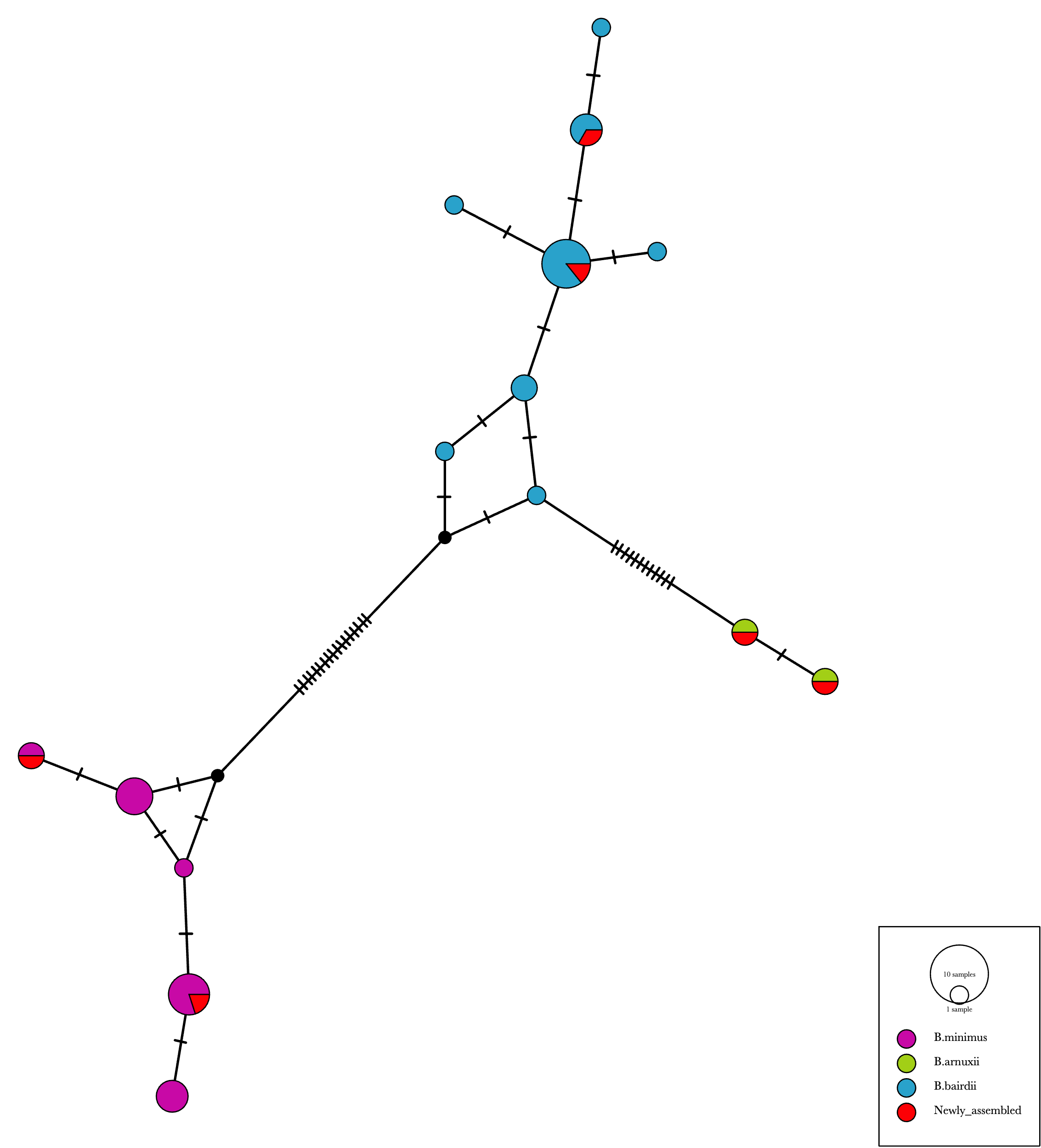


A


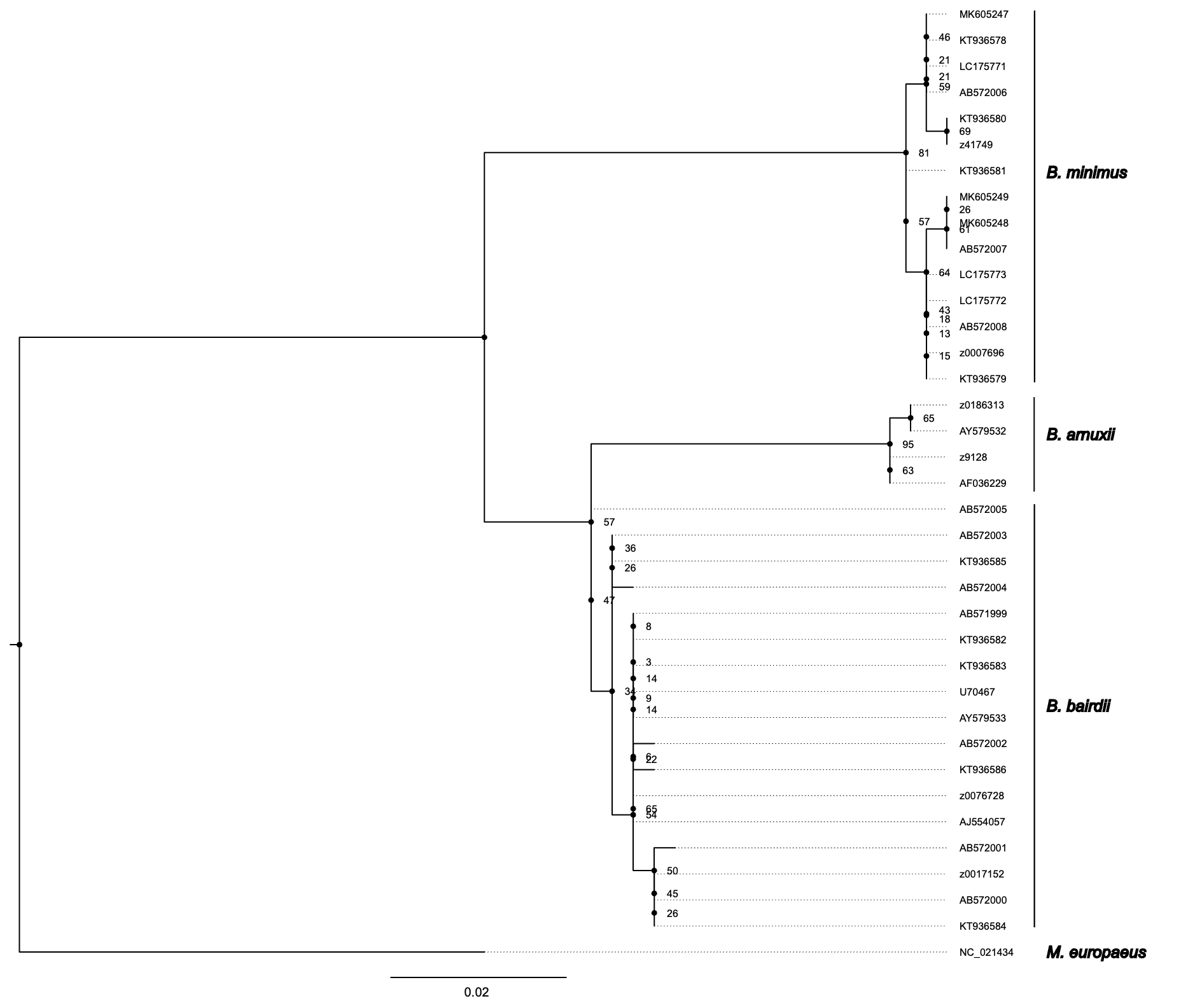


B

Supplementary Figure S5: A) Median-joining network of 35 D-Loop sequences representative of *B. bairdii*, *B. arnuxii* and *B. minimus* haplotypes. Newly assembled mtDNA by this study are colored red. Hash marks represent single fixed differences between haplotypes. B) Maximum likelihood tree of the 35 D-loop sequences rooted with *M. europaeus* as an outgroup.


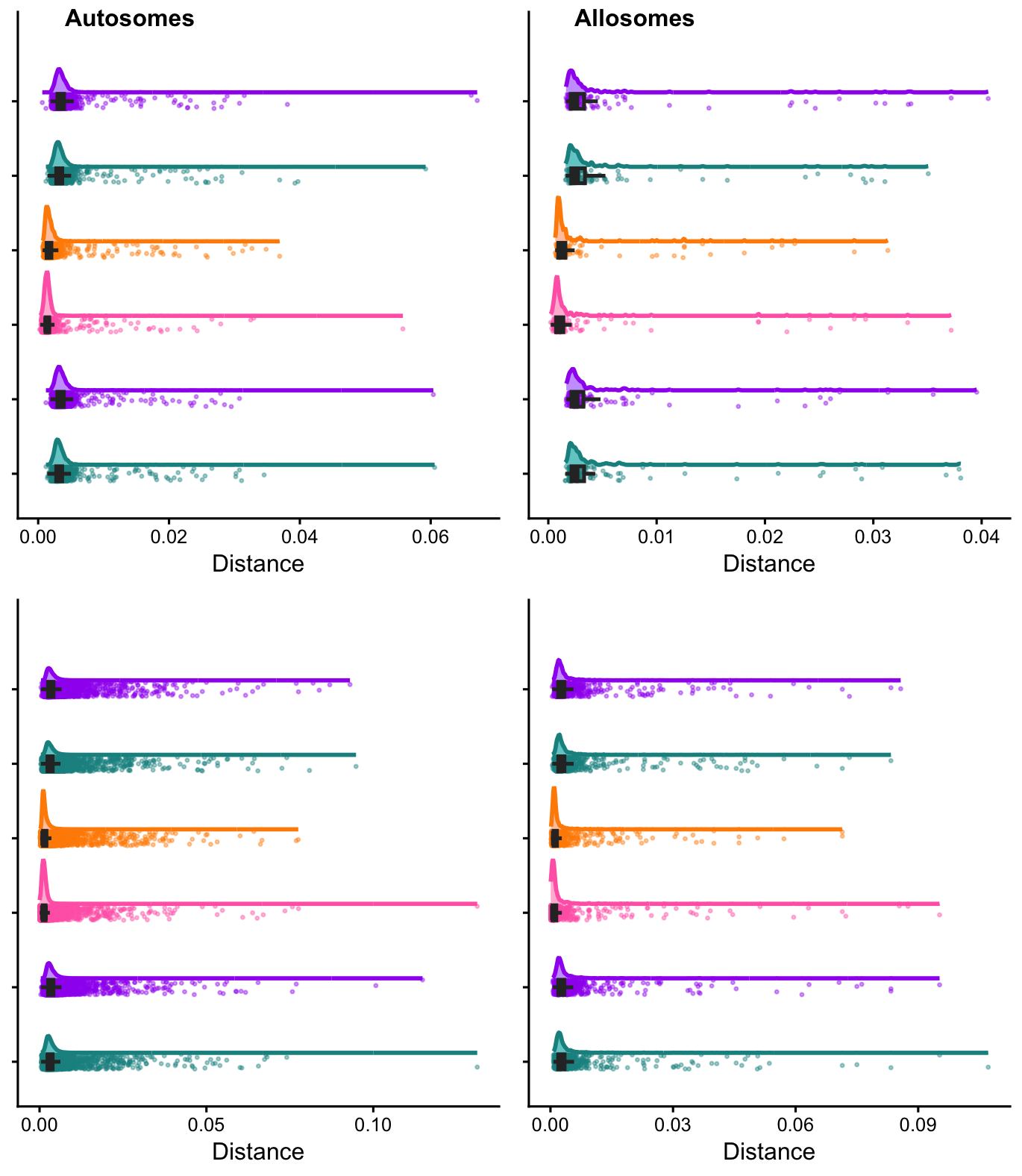

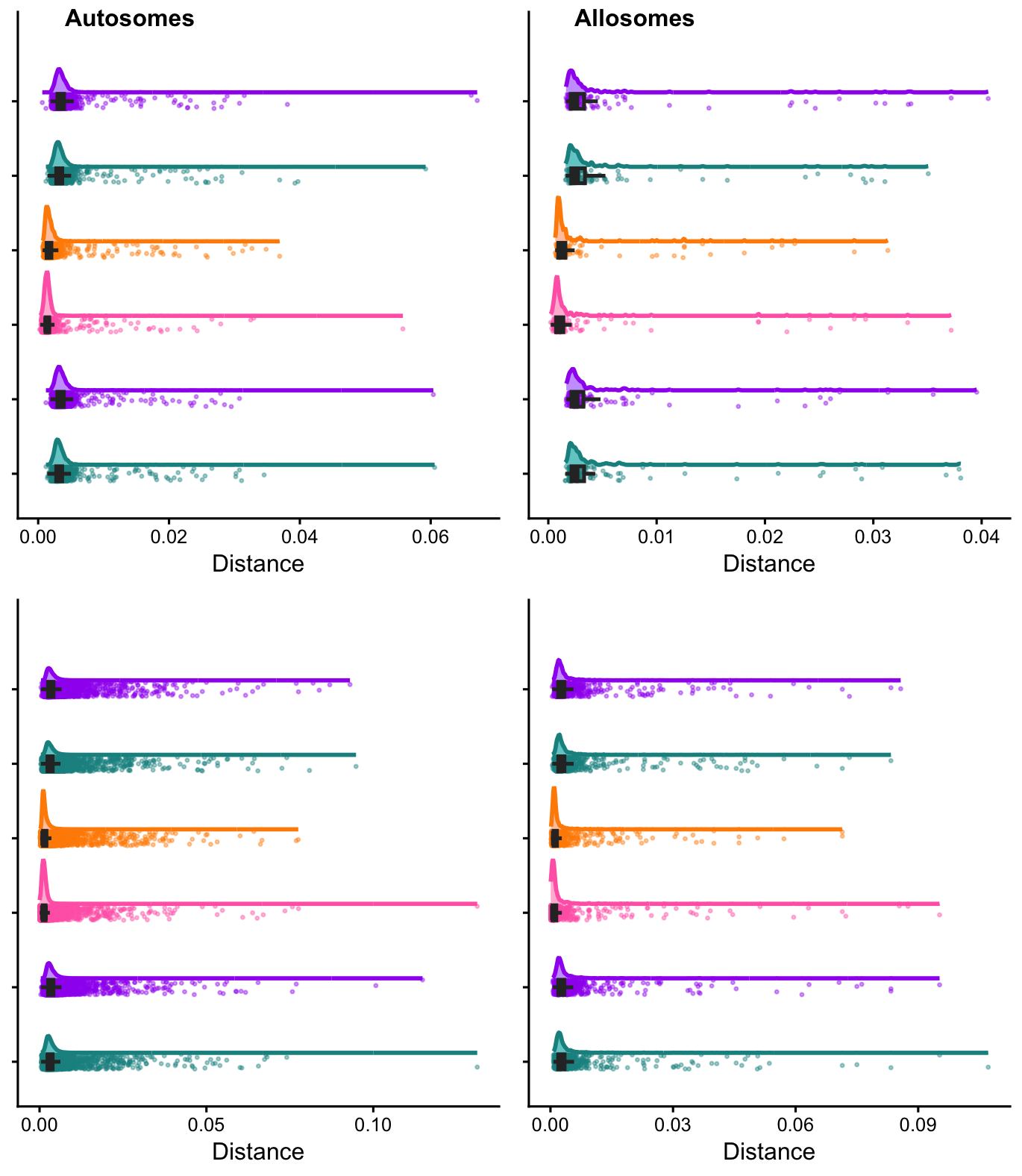

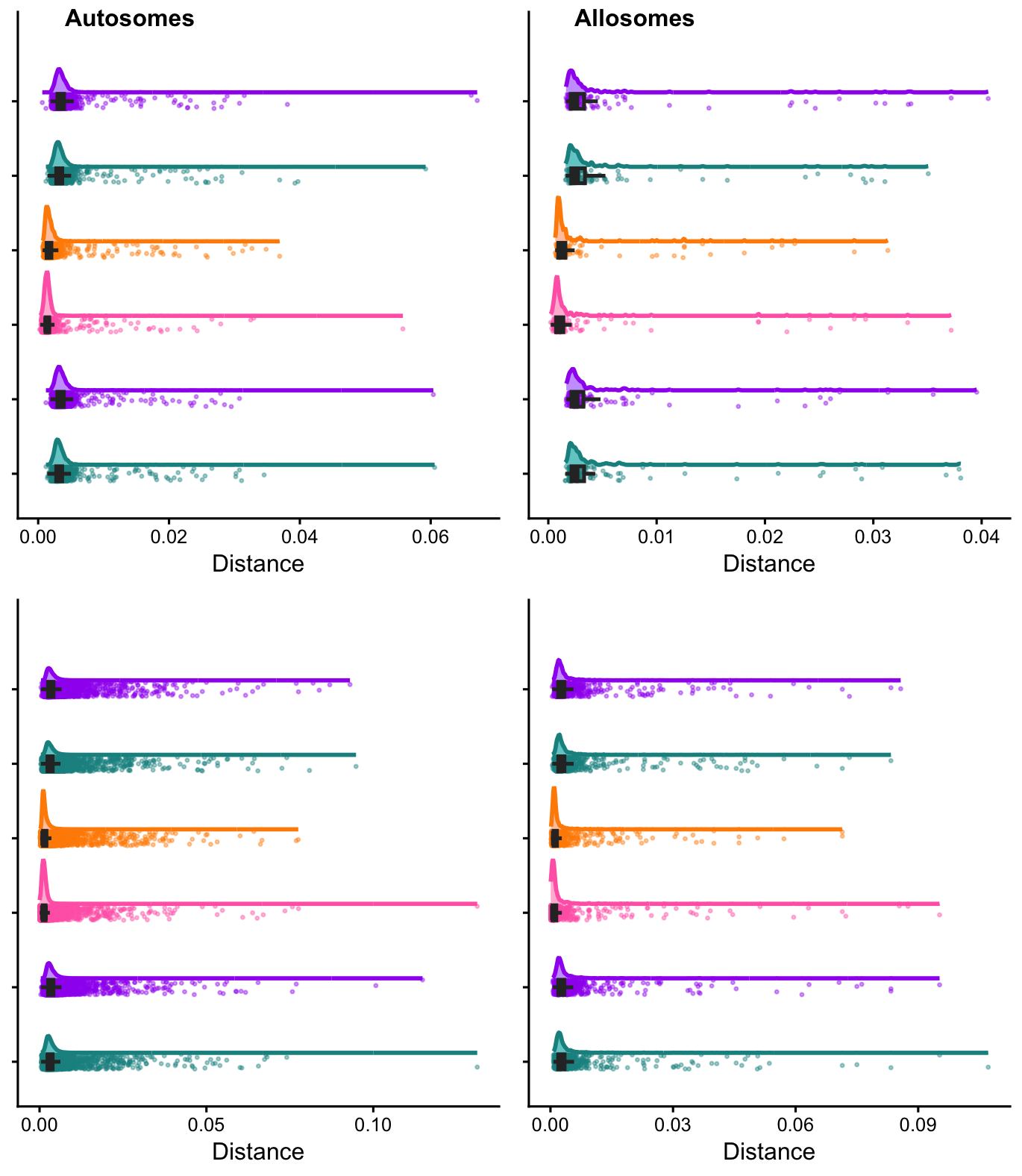


Distance (%)

Distance (%)

Supplementary Figure S6: Genetic distance among *Berardius* beaked whales using non-overlapping repeat masked autosomal (left) and allosomal (right) 1 Mb (top) and 100 kb (bottom) sliding window percent pairwise distances. The legend indicates two species included in a comparison. The top purple and cyan colored raincloud plots in each panel include *B.minimus* sample z0041749, and the bottom two include *B. minimus* sample z0007969.


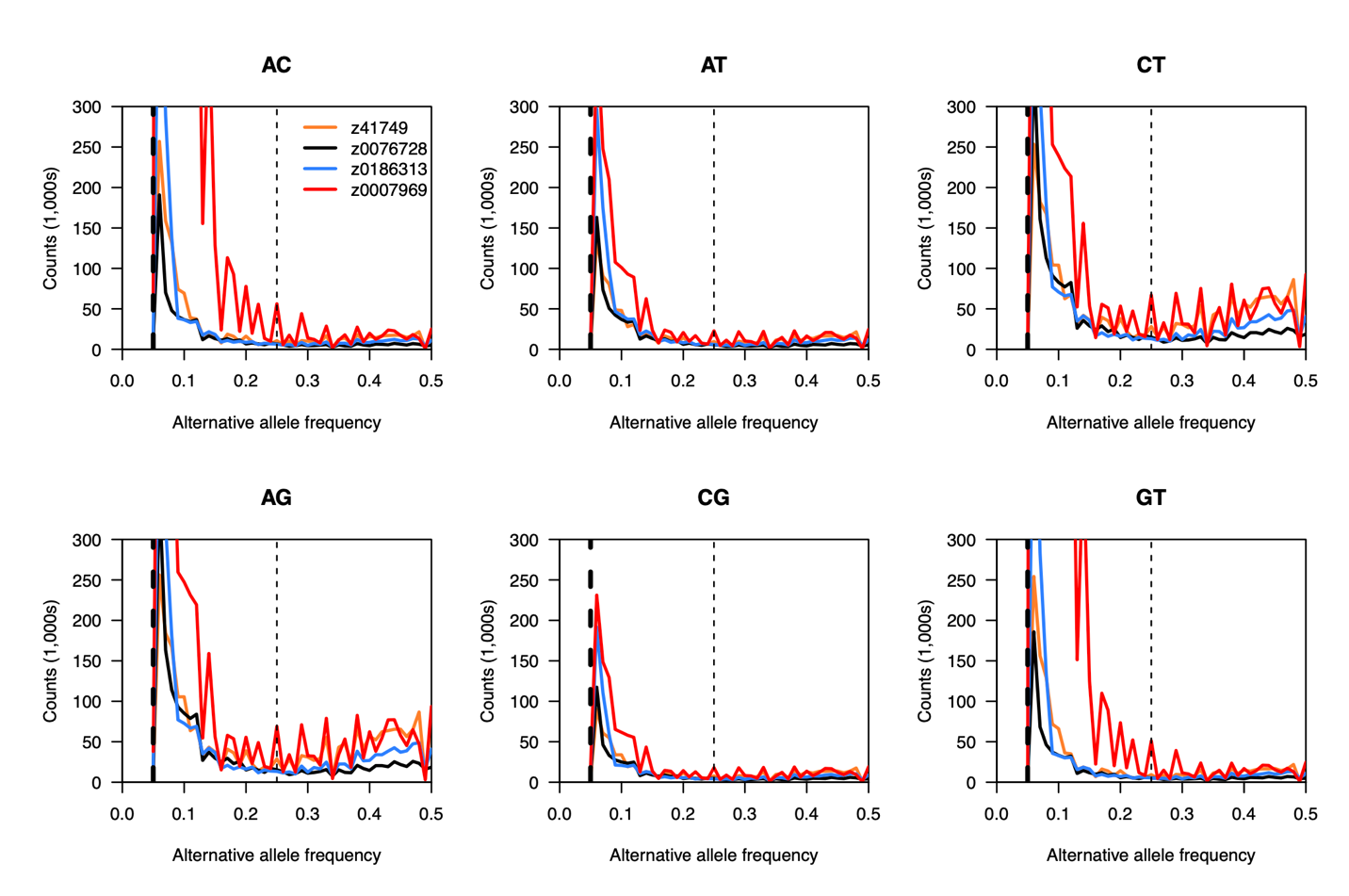


Supplementary Figure S7: Counts of alternative alleles separated based on their alternative allele frequencies showing the lower coverage *B. minimus* sample z0007969 had clearly elevated levels of heterozygous base calls at lower alternative allele frequencies.
